# Confirmation of the stereochemistry of spiroviolene

**DOI:** 10.3762/bjoc.20.77

**Published:** 2024-04-18

**Authors:** Yao Kong, Yuanning Liu, Kaibiao Wang, Tao Wang, Chen Wang, Ben Ai, Hongli Jia, Guohui Pan, Min Yin, Zhengren Xu

**Affiliations:** 1 State Key Laboratory of Natural and Biomimetic Drugs, School of Pharmaceutical Sciences, Peking University, Beijing 100191, China; Ningbo Institute of Marine Medicine, Peking University, Ningbo 315010, China; 2 State Key Laboratory of Microbial Resources, Institute of Microbiology, Chinese Academy of Sciences, Beijing 100101, China; University of Chinese Academy of Sciences, Beijing 100049, China; 3 School of Medicine, Yunnan University, 2 North Cui Hu Road, Kunming 650091, China

**Keywords:** boron migration, diterpene, spiroviolene, stereochemistry

## Abstract

We confirm the previously revised stereochemistry of spiroviolene by X-ray crystallographically characterizing a hydrazone derivative of 9-oxospiroviolane, which is synthesized by hydroboration/oxidation of spiroviolene followed by oxidation of the resultant hydroxy group. An unexpected thermal boron migration occurred during the hydroboration process of spiroviolene that resulted in the production of a mixture of 1α-hydroxyspiroviolane, 9α- and 9β-hydroxyspiroviolane after oxidation. The assertion of the *cis*-orientation of the 19- and 20-methyl groups provided further support for the revised cyclization mechanism of spiroviolene.

## Introduction

Terpenes represent one of the most fascinating families of natural products due to their structural complexity and diversity, as well as their indispensable biological functions that would be potentially applied as fragrances, pharmaceuticals etc. Until now, more than 80,000 terpenoid structures have been reported, which are found in all domains of life [1–3]. Despite their remarkable chemodiversity, the biosynthetic logic of terpenes is straightforward [4]. All terpenes are originated from two key C5 building blocks, namely isopentenyl pyrophosphate (IPP) and dimethylallyl pyrophosphate (DMAPP), which are biosynthesized via either the methylerythritol phosphate (MEP) pathway or the mevalonic acid (MVA) pathway by using the primary metabolites. Different numbers of IPP and DMAPP are assembled by prenyltransferases to afford oligoprenyl pyrophosphates, such as farnesyl pyrophosphate (FPP, 3 × C5) and geranylgeranyl pyrophosphate (GGPP, 4 × C5), with varied C5 units. The linear oligoprenyl pyrophosphates are typically converted by terpene synthases in a chemo- and stereoselective process to form complex terpene skeletons, normally with multiple stereocenters. In this context, the 3D-defined cyclization products retain the rich information of the complex cyclization process. Thus, assignment of the stereochemistry of the terpene skeleton with high confidence is crucial for proposing a reasonable cyclization mechanism [5].

**Figure 1:**
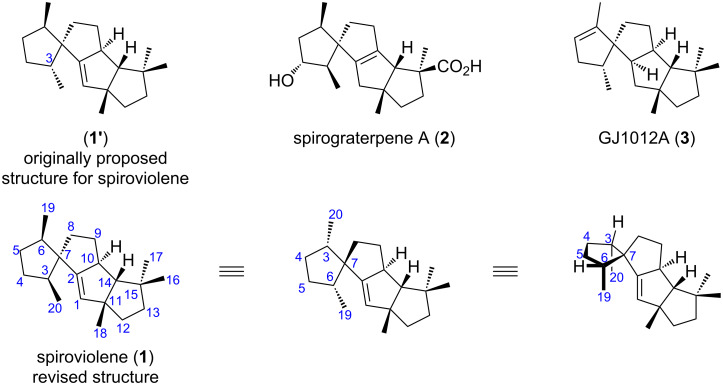
Structures of spiroviolene and related natural products.

Spiroviolene (**1**, Figure 1) was identified by Dickschat and co-workers as a nascent cyclization product of spiroviolene synthase (SvS), the coding gene of which was cloned from *Streptomyces violens* NRRL ISP-5597 [6]. Its unique spiro-fused linear triquinane to cyclopentane skeleton, as well as its stereochemistry, was originally elucidated as **1'** as shown in Figure 1, on the basis of detailed analysis of 1D and 2D NMR spectroscopy. Spiroviolene was also found to be produced by several bacterial strains harboring SvS homologs [6–7], as well as putative ancestors of SvS generated by ancestral sequence reconstruction [8–9]. Related natural products with the same 5-5-5-5 tetracyclic ring system, including spirograterpene A (**2**) from *Penicillium granulatum* MCCC 3A00475 [10], and GJ1012A (**3**) from an engineered *E. coli* strain harboring FgGS (FgJ07623) cloned from *Fusarium graminearum* GJ1012 [11], have been reported almost at the same time. The discrepancy of the stereochemistry at C3 between **1'** and **2** was first noticed by Snyder and co-workers [12]. The conserved stereochemistry of spiroviolene and **2** at C3 was later confirmed by the conversion of a synthetic intermediate of **2** to spiroviolene. By taking advantage of the DFT transition state analysis of the hydroboration reaction of a key intermediate, as well as NOE correlation analysis of the resultant product, Snyder and co-workers have reassigned the right structure of spiroviolene to **1**. However, direct evidences such as single-crystal X-ray diffraction results were not reported in their study.

**Scheme 1:**
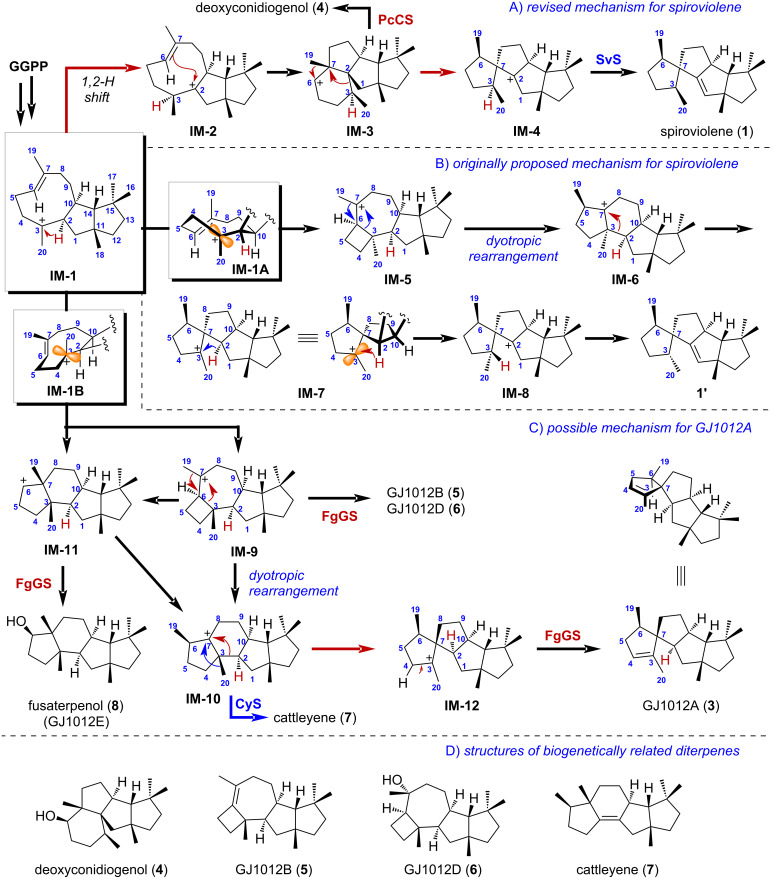
Possible cyclization mechanisms for spiroviolene (**1**) and related natural products. A) Revised cyclization mechanism for **1** that is related to deoxycondiogenol (**4**). B) Originally proposed cyclization mechanism for the misassigned structure **1'**. C) Possible cyclization mechanism for GJ1012A (**3**). D) Structures of biogenetically related diterpenes during the proposed cyclization process.

The reassignment of the stereochemistry at C3 has resulted in the revision of the proposed cyclization mechanism [12–14]. The revised mechanism resembled the cyclization process for the formation of deoxyconidiogenol (**4**, Scheme 1A) by several terpene cyclases from fungus (PcCS, PchDS, PrDS) [15–16], which involves a 1,11-10,14 cyclization of GGPP, followed by 1,2-alkyl shift and a 2,10-cyclization, to give the key C3 cationic intermediate **IM-1**. A key 1,2-hydride shift from C2 to C3, which was observed in the isotope labeling experiments [6], followed by a 2,7-cyclization, afforded C6 cationic intermediate **IM-3** with cyclopiane skeleton. Quench of the cation **IM-3** with water would give **4**, while upon two 1,2-alkyl shifts of **IM-3**, followed by deprotonation of cation **IM-4**, would give spiroviolene (**1**).

On the other hand, the originally proposed cyclization mechanism (Scheme 1B) involves a 3,6-cyclization of cation **IM-1** through a conformation shown as **IM-1A** to generate cation **IM-5**, which was proposed to undergo a dyotropic rearrangement, followed by a 1,2-alkyl shift of cation **IM-6** to yield the spirocyclic cation **IM-7**. A key 1,3-hydride shift of **IM-7** from the β-face, followed by deprotonation of the formed C2-cation **IM-8**, would deliver the originally proposed structure **1'** [6]. However, no related natural products that would be derived from the intermediates of this pathway have been found so far.

A third cyclization mechanism (Scheme 1C) leading to the same spirocyclic skeleton as spiroviolene with an altered stereochemistry at C7 found in GJ1012A (**3**) could be proposed [11,17–18]. A 3,6- or 3,7-cyclization of cation **IM-1** through a conformation shown as **IM-1B** with β-oriented 20-methyl group, would generate either **IM-9** or **IM-11** cations. A direct dyotropic rearrangement, or two stepwise 1,2-alkyl migrations of **IM-9**, are possible pathways en route to cation **IM-10**. The presence of these intermediates **IM-9, -10, -11** could be inferred by the identification of GJ1012B/D (**5/6**, Scheme 1D) [11], cattleyene (**7**) [19–20], and fusaterpenol (**8**, GJ1012E) [17]. A similar 1,2-alkyl shift of **IM-10**, followed by deprotonation of the formed spirocyclic cation **IM-12**, afforded **3**. Although previous isotope labeling experiments did not support this pathway for spiroviolene cyclization, it should be noted that a subtle alteration of stereochemical assignment of spiroviolene would have consequences for a different mechanistic proposal. We herein report the production of spiroviolene (**1**) in a heterologous host by taking advantage of an artificial isopentenol utilization pathway [21–26], and confirm its stereochemistry by X-ray crystallography using a hydrazone derivative of **1**.

## Results and Discussion

**Figure 2:**
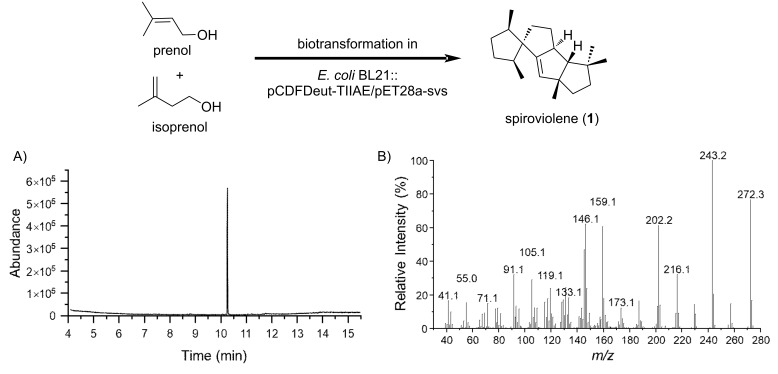
Heterologous production of spiroviolene using the isopentenol utilization pathway. A) Gas chromatogram of the EtOAc extract of the fermentation broth. B) EI mass spectrum of spiroviolene.

Our work commenced with the heterologous production of spiroviolene by *E. coli* using a recently developed isopentenol utilization pathway for the efficient supply of two C5 precursors for terpene biosynthesis (Figure 2) [21–26]. In this artificially generated pathway, DMAPP and IPP could be easily generated from prenol and isoprenol, respectively, by the effect of two kinases, such as hydroxyethylthiazole kinase from *E. coli* (*Ec*ThiM) and isopentenyl phosphate kinase from *Methanocaldococcus jannaschii* (*Mj*IPK). Thus, we have cloned genes coding *Ec*ThiM, *Mj*IPK, isopentenyl diphosphate isomerase of *E. coli* (IDI), farnesyl diphosphate synthase of *E. coli* (IspA) and geranylgeranyl diphosphate synthase of *Pantoea agglomerans* (CrtE) into the multiple cloning site-2 of pCDFDeut-1 to give pCDFDeut-TIIAE for GGPP production. Also, we have cloned the SvS-coding gene directly from *Streptomyces violens* CGMCC 4.1786 (= NRRL ISP-5597) into pET28a to give pET28a-svs. The resultant two plasmids were then co-transformed into commercially available *E. coli* BL21(DE3) for diterpene production. Spiroviolene could be produced by feeding prenol and/or isoprenol to the fermentation broth after *E. coli* being induced by IPTG, and fermented at 18 °C for 72 h. In our hand, feeding a mixture of prenol and isoprenol in a 1:1 ratio would give the best yield of spiroviolene. GC–MS analysis (Figure 2A) of the EtOAc extract of the fermentation broth gave a single peak, whose EIMS spectrum (Figure 2B) matches that of spiroviolene. We then carried out a large-scale fermentation using 2 L-shake flasks, and each flask contains 1.0 L of modified TB medium. We have isolated 201 mg (40 mg/L) of spiroviolene from 5.0 L of the fermentation broth. The physicochemical data of the isolated material are consistent with those reported for spiroviolene [6].

With sufficient amount of spiroviolene in hand, we next attempted to obtain a crystalline compound suitable for X-ray diffraction by introducing functional groups (e.g., a hydroxy group, or a keto group) for further derivatization. Spiroviolene was not transformed when subjected to conditions for allylic oxidation (SeO_2_) even at elevated temperature [27], and the starting material was fully recovered.

**Scheme 2:**
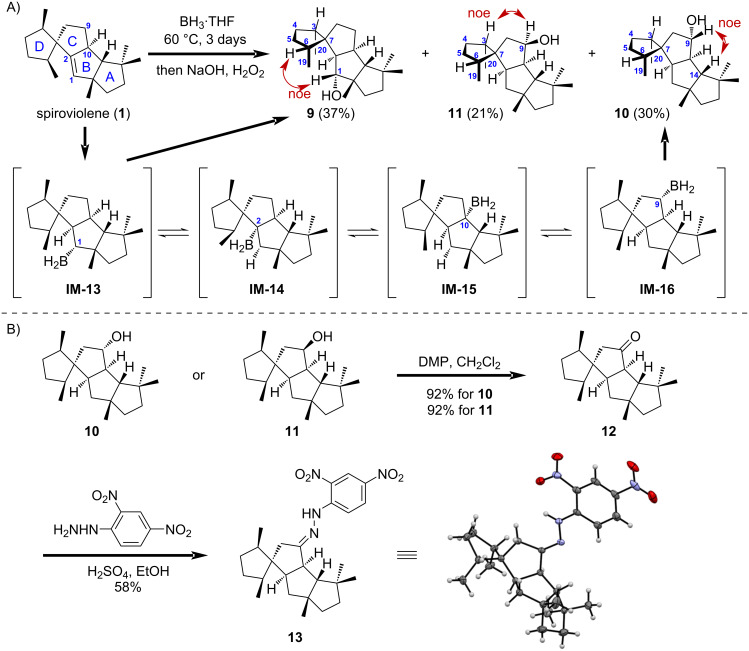
Derivatization of spiroviolene for X-ray crystallography. A) Hydroboration/oxidation reaction of spiroviolene involving a borane migration process. B) Synthesis of the hydrazone derivative of spiroviolene for single-crystal X-ray diffraction.

We have also tried hydroboration/oxidation conditions for transforming the double bond in a congested environment of spiroviolene (Scheme 2). Low conversion was observed when a tetrahydrofuran (THF) solution of **1** was treated with BH_3_·THF at ambient temperature. The hydroboration reaction could be driven to synthetically useful yield when **1** was directly dissolved in 1 M BH_3_·THF in THF, and heated at 60 °C for 3 days. After oxidative treatment of the resultant alkylborane products with NaOH/H_2_O_2_, we have obtained three derivatives **9**–**11** with one hydroxy group in 37%, 30% and 21% isolated yield, respectively, as well as recovery of 10% of the starting material. After detailed analysis of the NMR spectra, we have found that besides the normal hydroboration/oxidation product 1α-hydroxyspiroviolane (**9**), 9α- (**10**) and 9β-hydroxyspiroviolane (**11**) resulting from a formal boration at the homoallylic C9 position were also produced. The stereochemistry of the newly generated stereocenters was elucidated on the basis of their NOESY spectra. Thus, the key NOE correlations of H-1/H-6, H-1/H_3_-19, and H-2/H_3_-20 of **9** allowed to assign the 1-OH to be α-oriented, while correlations of H-9/H-14 of **10**, and H-9/H-3 of **11**, supported the assignment of 9α- and 9β-oriented hydroxy groups, respectively.

The formation of all three products **9–11** can be explained as follows (Scheme 2A) [28–31]: Due to the favorable formation of *cis*-5,5-fused B/C ring system, the borane reagent is preferred to approach the double bond of **1** from the α-face, to give either a secondary 1-organoborane intermediate **IM-13**, or a tertiary 2-organoborane intermediate **IM-14**. Oxidation of **IM-13** with H_2_O_2_/NaOH could stereoselectively furnish the normal hydroboration/oxidation product **9**. On the other hand, the unstable tertiary 2-organoborane **IM-14** would undergo two consecutive thermal 1,2-boron migrations to give the 9-organoborane intermediate **IM-16** probably through borane–olefin complexes. The suprafacial nature of the boron migration allowed the boron to be α-oriented in intermediate **IM-16**, which would give **10** with retention of the configuration after NaOH/H_2_O_2_ oxidation. The formation of a significant amount of the C-9 epimerization product **11** might be explained by a competing radical oxidation of the organoborane intermediate **IM-16** by oxygen when the oxidation step is opened to air [32–33]. On the other hand, an alternative mechanism for the formation of **11** involving the oxidation of the 9-*epi*-**IM-16** organoborane intermediate, which was formed by elimination of BH_3_ from **IM-16** followed by re-addition of BH_3_ from the opposite β-face to the proposed C8–C9 double bond intermediate, would also be possible.

To further advance the intermediate to crystalline hydrazone product (Scheme 2B), we have found that both **10** and **11** can be oxidized to the same 9-oxospiroviolane (**12**) in the same 92% isolated yield, hence confirming the structural assignment of **10** and **11**. By reacting with 2,4-dinitrophenylhydrazine [34], ketone **12** was further converted to hydrazone derivative **13**, which gave a brownish-yellow crystal suitable for X-ray diffraction [35]. The crystal structure of **13** clearly showed that the 19- and 20-methyl groups are *cis*-oriented in the D-ring which is consistent with that of spirograterpene A. This structural data reaffirms the revised structure of spiroviolene, and further support the unified cyclization process of fungi-derived deoxyconidiogenol and bacteria-derived spiroviolene by sharing the common C6-cation intermediate **IM-3** with cyclopiane skeleton (Scheme 1A).

## Conclusion

We have unambiguously confirmed the structural revision of spiroviolene with *cis*-oriented 19- and 20-methyl groups by obtaining a suitable crystal of the hydrazone derivative of 9-oxospiroviolane, which was synthesized by hydroboration/oxidation of spiroviolene followed by converting the resultant hydroxy group to a keto group, for single-crystal X-ray diffraction. The similar skeleton and conserved stereochemistry of spiroviolene and spirograterpene A therefore indicated that there must exist two terpene cyclases of different origins (Actinomycetes and Fungus) that were able to carry out similar chemical processes for the formation of the intriguing 5-5-5-5 tetracyclic ring system. Also, our study supports the proposed unified cyclization processes of spiroviolene and deoxyconidiogenol that bifurcate at the C6-cation intermediate **IM-3** with a cyclopiane skeleton. Thus, further mutational studies of these related terpene cyclases would give us more insights into the complex cyclization processes.

## Supporting Information

File 1 Materials, synthetic methods, and copies of NMR spectra for all compounds.

File 2 Crystallographic information file of compound **13**.

## Data Availability

All data that supports the findings of this study is available in the published article and/or the supporting information to this article.
