# Supplementary material for: Confirmation of the stereochemistry of spiroviolene
Source: Beilstein J Org Chem. 2024 Apr 18;20:852–8. doi: 10.3762/bjoc.20.77 (PMC11035986; doi:10.3762/bjoc.20.77)
Supplement: File 1 — Materials, synthetic methods, and copies of NMR spectra for all compounds. [file Beilstein_J_Org_Chem-20-852-s001.pdf]

BEILSTEIN JOURNAL OF ORGANIC CHEMISTRY

Supporting Information

for

Confirmation of the stereochemistry of spiroviolene

Yao Kong, Yuanning Liu, Kaibiao Wang, Tao Wang, Chen Wang, Ben Ai, Hongli Jia, Guohui Pan, Min Yin and Zhengren Xu

Beilstein J. Org. Chem. **2024,** *20,* 852–858. doi:10.3762/bjoc.20.77

Materials, synthetic methods, and copies of NMR spectra for all compounds

License and Terms: This is a supporting information file under the terms of the Creative Commons Attribution License (https://creativecommons.org/licenses/by/4.0). Please note that the reuse, redistribution and reproduction in particular requires that the author(s) and source are credited and that individual graphics may be subject to special legal provisions.
The license is subject to the *Beilstein Journal of Organic Chemistry* terms and conditions: (https://www.beilstein-journals.org/bjoc/terms)

Table of Contents

**Material and Methods**	S2–S3
**Derivatization of spiroviolene for X-ray diffraction**	S3–S4
**Tables S1–S2: Strains, plasmids, and primers used in this study**	S5
**Table S3: ^{1}H NMR data of spiroviolene (1)**	S6
**Table S4: ^{13}C NMR data of spiroviolene (1)**	S7
**Table S5: ^{1}H NMR data of compounds 9-11**	S8
**Table S6: ^{13}C NMR data of compounds 9-11**	S9
**Figure S1: Key ^{1}H-^{1}H COSY, HMBC and NOESY correlations of 1, 9–11**	S10
**Figures S2a–S2g: NMR spectra of spiroviolene (1) in C_6D_6**	S11–S17
**Figures S3a–S3g: NMR spectra of spiroviolene (1) in $CDCl_3$**	S18–S24
**Figures S4a–S4g: NMR spectra of compound 9 in $CDCl_3$**	S25–S31
**Figures S5a–S5g: NMR spectra of compound 11 in $CDCl_3$**	S32–S38
**Figures S6a–S6g: NMR spectra of compound 10 in $CDCl_3$**	S39–S45
**Figures S7a–S7c: NMR spectra of compound 12 in $CDCl_3$**	S46–S48
**Figures S8a–S8c: NMR spectra of compound 13 in $CDCl_3$**	S49–S51
**References**	S52

Materials and Methods

Bacterial strains, plasmids, chemicals

Strains, plasmids, and PCR primers used in this study are listed in Tables S1 and S2. PCR primers were purchased from Ruibiotech (Beijing). KOD One^{TM} PCR Master Mix (Toyobo Co., Ltd.), Gibson Assembly Kit (TransGen) were purchased from corresponding commercial suppliers and reactions were performed according to the manufacturer's protocols. DNA gel extraction and plasmid preparation kits were purchased from TransGen. DNA sequencing was conducted by Majorbio. Other common chemicals, bio-chemical, and media components were purchased from standard commercial sources.

General procedures

E. coli strains harboring plasmids were grown in lysogeny broth (LB) with appropriate antibiotics. *Streptomyces violens* CGMCC 4.1786 was cultivated on solid ISP4 medium for sporulation. Actinomycetes were cultivated in liquid tryptic soy broth (TSB) at 28 ^{o}C to prepare the mycelium for genomic DNA (gDNA) isolation. Isolation of gDNA from Actinomycetes strains were performed using the salting out protocol.[1]

IR spectra were collected with a Nicolet Nexus 470 spectrometer. Optical rotation was recorded with a Rudolph Autopol VI digital Polarimeter. GC–MS data were collected on an Agilent 7890A/5975C GC–MS apparatus with a DB-5MS column (30 m × 0.25 mm × 0.25 μm). All ^{1}H, ^{13}C, and 2D-NMR (HSQC, ^{1}H-^{1}H COSY, HMBC, NOESY) spectra were collected at room temperature (25 ^{o}C) with a Bruker AVANCE III 400 at 400 MHz for ^{1}H and 100 MHz for ^{13}C nuclei, or a Bruker Avance III 600 at 600 MHz for ^{1}H and 150 MHz for ^{13}C nuclei. Chemical shifts were calibrated with the solvent residue signals (δ $CDCl_3$: 7.26, 77.16; C_6D_6: 7.16, 128.06 for ^{1}H and ^{13}C NMR spectra). HR-ESI-MS data were acquired on a Waters XEVO G2 QTOF instrument. Melting point was recorded with a BÜCHI M-560 melting point apparatus.

Single-crystal X-ray diffraction data collection of **13** was measured on a Rigaku Oxford Diffraction XtaLAB Synergy four-circle diffractometer equipped with a microfocus Cu Kα X-ray source (1.54184 Å, PhotonJet-R 1200W) and a HyPix-6000C area detector. The sample crystal was cooled to 100 K using a cold nitrogen stream (Cobra by Oxford Cryosystems). Date reduction, cell refinement and experimental absorption correction were performed in CrysAlisPro[2]. Crystal data: $C_{26}H_{36}N_4O_4$, M = 468.59, monoclinic, space group C 2y; unit cell dimensions were determined to be a = 18.5331 (3) Å, b = 8.16904 (10), c = 33.4838 (4), α = 90°, β = 102.1517 (13) °, γ = 90°, V = 4955.78 (12) Å^3, Z = 8, Dx = 1.256 g/cm^3, $F(000)$ = 2016.0, μ (Cu Kα) = 1.542 mm^{-1}. 31246 reflections were collected until θ_{max} = 72.095°, in which independent unique 9556 reflections were observed [$F^2 > 4\sigma(F^2)$]. The final refinement of all data gave R = 0.0380, wR_2 = 0.0342, and S = 1.024. Structure solution, refinement, and data output were performed with the OLEX2 program package[3] using SHELXL-2014[4] for the refinement. Multi-scan method was used for the absorption correction. Structures were solved by direct methods and refined against F^2 by full-matrix least-squares. All non-hydrogen atoms were refined anisotropically. Hydrogen atoms were generated geometrically at idealized position and constrained to ride on their parent. The crystallographic data for this paper were deposited in CCDC database with codes of 2274944.

Cloning SvS-coding gene from *Streptomyces violens*

To construct plasmid for production of terpene cyclase SvS for spiroviolene production, the region coding for *svS* was PCR amplified from the gDNA of *S. violens* CGMCC 4.1786 using primers as listed in Table S2. The gel-recovered DNA fragment was clone into pET28a using Gibson Assembly Kit to give pET28a-svS, which was co-transformed with pCDFDuet-TIIAE into E. coli BL21(DE3) for spiroviolene production.

Production of spiroviolene

A single colony of the transformant was collected from the plate and inoculated into 100 mL of LB medium containing streptomycin (50 μg/mL) and kanamycin (50 μg/mL). The seed culture was allowed to shake at 37 °C overnight, and 10 mL of which was inoculated into a 2L-flask containing 1L of modified TB medium (12 g/L tryptone, 24 g/L yeast extract and 20 g/L glycerol). The resultant culture was allowed to grow at 37 °C with a shaking speed of 200 rpm until OD_{600} reached 0.6. Isopropyl-β-D-thiogalactopyranoside (IPTG, 0.5 mM), prenol (400 μL/L) and isoprenol (400 μL/L) were added to the culture. The resultant culture was fermented at 18 °C with a speed of 200 rpm for 72 h.

Purification of spiroviolene

EtOAc (500 mL) was added to the fermentation broth (1 L), and the mixture was filtered through a pad of Celite. The separated aqueous phase was extracted with EtOAc (2 × 500 mL). The combined organic layers were dried over Na_2SO_4, and the solvent was evaporated under reduced pressure. The crude product was purified by flash chromatography on silica gel, and eluted with petroleum ether (PE) to yield spiroviolene (**1**) as a colorless oil (40 mg/L). R_f = 0.97 (pure PE); $[\alpha]^{20}_D$ -4.5 (*c* 0.2, C_6D_6) ($[\alpha]_D$ -5.6 (*c* 0.2, C_6D_6)[5]; $[\alpha]_D$ -5.4 (*c* 0.2, C_6D_6)[6]); IR (neat) ν_{max} 2927, 2865, 1462 cm^{-1}; ^1H- and ^{13}C-NMR data are listed in Table S3-4.

Derivatization of spiroviolene for X-ray diffraction

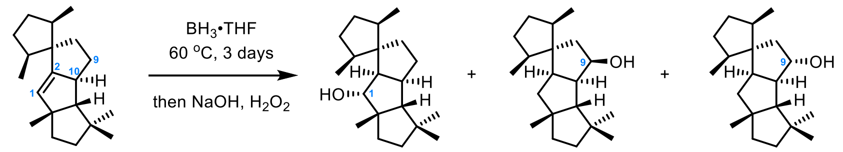

spiroviolene (**1**) **9** (37%) **11** (21%) **10** (30%)

Spiroviolene (**1**, 72.0 mg, 0.26 mmol, 1.0 equiv) was dissolved in BH_3·THF complex (1.0 M in THF, 2.0 mL, 7.7 equiv), and the reaction mixture was heated at 60 °C under nitrogen atmosphere for 3 days. To the resultant reaction mixture at room temperature was added 20% aq. solution of NaOH (5.0 mL) and 30% aq. solution of H_2O_2 (5.0 mL). The reaction mixture was allowed to stir at room temperature for 2 h. The mixture was extracted with CH_2Cl_2 (15 mL × 3). The combined organic layers were dried over Na_2SO_4 and evaporated to dryness. The resultant residue was purified with silica gel chromatography (PE/EtOAc = 20:1) to give recoved spiroviolene (7.0 mg, 10%), 1α-hydroxy-spiroviolane **9** (28.0 mg, 37%), 9β-hydroxy-spiroviolane **11** (16.0 mg, 21%) and 9α-hydroxy-spiroviolane **10** (23.0 mg, 30%), respectively.

1α-Hydroxyspiroviolane (9): Colorless oil; R_f = 0.64 (PE/EtOAc = 10:1); $[\alpha]^{20}_D$ -60 (*c* 0.1, $CHCl_3$); IR (neat) ν_{max} 3501, 2952, 2872, 1464, 1378 cm^{-1}; ^1H-NMR and ^{13}C-NMR data are listed in Tables S5-6; HR-ESI-MS *m*/*z* 289.2533 $[M - H]^-$ (calcd. for $C_{20}H_{33}O^-$, 289.2537).

9β-Hydroxyspiroviolane (11): Colorless oil; R_f = 0.48 (PE/EtOAc = 10:1); $[\alpha]^{20}_D$ -46 (*c* 0.2, $CHCl_3$); IR (neat) ν_{max} 3414, 2947, 2868, 1460, 1378 cm^{-1}; ^1H-NMR and ^{13}C-NMR data are listed in Tables S5-6; HR-ESI-MS *m*/*z* 289.2537 $[M - H]^-$ (calcd. for $C_{20}H_{33}O^-$, 289.2537).

9α-Hydroxyspiroviolane (10): Colorless oil; R_f = 0.36 (PE/EtOAc = 10:1); $[\alpha]^{20}_D$ -49 (*c* 0.1, $CHCl_3$); IR (neat) ν_{max} 3341, 2949, 2868, 1459, 1378 cm^{-1}; ^1H-NMR and ^{13}C-NMR data are listed in Tables S5-6; HR-ESI-MS *m*/*z* 289.2537 $[M - H]^-$ (calcd. for $C_{20}H_{33}O^-$, 289.2537).

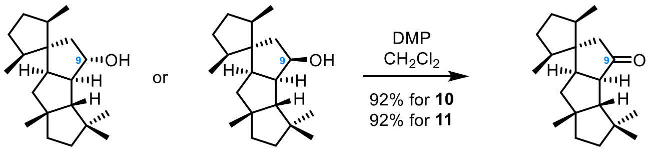

10 **11** **12**

9-Oxospiroviolane (12): To a stirring solution of 9α-hydroxy-spiroviolane (**10**, 6.0 mg, 0.021 mmol, 1.0 equiv) in CH_2Cl_2 (2.0 mL) at room temperature was added Dess-Martin Periodinane (10.7 mg, 0.025 mmol, 1.2 equiv). The reaction mixture was stirred at room temperature until TLC analysis showed the full consumption of the starting material. The reaction was quenched by addition of sat. aq. $NaHCO_3$ solution (2.0 mL), and the mixture was extracted with CH_2Cl_2 (5 mL × 3). The combined organic layers were dried over Na_2SO_4, and evaporated to dryness. The crude product was purified with silica gel chromatography (CH_2Cl_2/MeOH = 25:1) to yield **12** (5.6 mg, 92%) as a colorless oil. R_f = 0.53 (PE/EtOAc = 10:1); $[\alpha]^{20}_D$ +25 (*c* 0.1, $CHCl_3$); IR (neat) ν_{max} 2953, 2869, 1740, 1460, 1379 cm^{-1}; ^{1}H-NMR (400 MHz, $CDCl_3$) *δ* 2.73 (dt, *J* = 11.1, 7.5 Hz, 1H), 2.38 (d, *J* = 7.2 Hz, 1H), 2.14-2.13 (m, 2H), 2.11-2.05 (m, 1H),1.95-1.91 (m, 1H), 1.91-1.82 (m, 2H), 1.77-1.66 (m, 2H), 1.62-1.58 (m, 2H), 1.50-1.44 (m, 4H), 1.30-1.26 (m, 1H),1.10 (s, 3H), 1.07 (s, 3H), 1.07 (d, *J* = 7.3 Hz, 3H), 1.01 (d, *J* = 7.2 Hz, 3H), 0.95 (s, 3H); ^{13}C-NMR (100 MHz, $CDCl_3$) *δ* 223.3, 63.7, 58.8, 52.7 (2C), 52.1, 45.6, 45.0, 43.0, 42.54, 42.52, 42.0, 40.5, 32.4, 31.8, 31.1, 30.7, 26.2, 20.7, 18.0; HR-ESI-MS *m/z* 289.2535 [M + H]$^+$ (calcd. for $C_{20}H_{33}O^+$, 289.2526).

9β-Hydroxyspiroviolane (**11**) was also transformed into **12** in the same yield (92%) by using the same conditions as that of **10**.

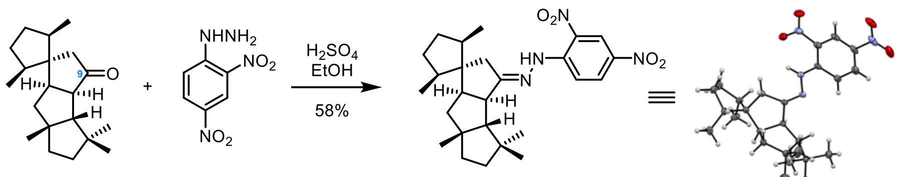

12 **13**

Compound 13[7]: Solid 2,4-dinitrophenylhydrazine (55.5 mg, 0.28 mmol, 4.0 equiv) and conc. H_2SO_4 (2.0 mL) were mixed and stirred at room temperature for 10 min to dissolve all the solid. To the resultant reaction mixture with stirring was added a solution of **12** (21.0 mg, 0.07 mmol, 1.0 equiv) in 95% EtOH (2.0 mL) dropwise. The reaction mixture was allowed to stir at room temperature for 30 min until the precipitation of brownish-yellow solid was observed. The mixture was filtered off, and the solid was washed with EtOH (~2 mL). The solid was desiccated to afford **13** (19.0 mg, 0.04mmol, 58%) as a brownish-yellow solid. R_f = 0.53 (PE/EtOAc = 10:1); m.p. 164-168 °C (brownish-yellow needle crystal, PE/CH_2Cl_2, 5:1); $[\alpha]^{20}_D$ +133 (*c* 0.1, $CHCl_3$); IR (neat) ν_{max} 3315, 2954, 2919, 2869, 1619, 1591, 1337 cm^{-1}; ^{1}H-NMR (400 MHz, $CDCl_3$) *δ* 10.75 (br s, 1H), 9.13 (d, *J* = 2.6 Hz, 1H), 8.31 (dd, *J* = 9.6, 2.6 Hz, 1H), 7.94 (d, *J* = 9.6 Hz, 1H), 2.95 (d, *J* = 6.5 Hz, 1H), 2.65 (dt, *J* = 11.1, 7.3 Hz, 1H), 2.44-2.29 (m, 2H), 2.19-2.09 (m, 2H), 1.92-1.87 (m, 2H), 1.86-1.79 (m, 1H), 1.68-1.56 (m, 3H), 1.57-1.45 (m, 4H), 1.25-1.19 (m, 1H), 1.16 (s, 3H), 1.11 (s, 3H), 1.07 (d, *J* = 7.2 Hz, 3H), 1.06 (s, 3H), 1.02 (d, *J* = 7.1 Hz, 3H); ^{13}C-NMR (100 MHz, $CDCl_3$) *δ* 171.1, 145.1, 137.7, 130.2, 128.9, 123.7, 116.4, 66.5, 56.0, 53.5, 52.8, 47.0, 44.7, 42.9, 42.8, 42.6 (2C), 42.1, 40.7, 32.4, 31.7, 31.5, 31.2, 26.3, 20.5, 18.4; HR-ESI-MS *m/z* 469.2808 [M + H]$^+$ (calcd. for $C_{26}H_{37}N_4O_4^+$, 469.2809).

Table S1. Strains and plasmids used in this study

Plasmids/Strains	Relevant Characteristic	Source
pCDFDeut-TIIAE	pCDFDuet-1 derived plasmid for the production of GGPP, harboring gene *Ec*THIM, *Mj*IPK, *idi*, *ispA* and *crtE*	This study
pET28a-svs	pET28a(+) derived plasmid for the production of spiroviolene, harboring SvS-coding gene	This study
Streptomyces violens CGMCC 4.1786	Bacteria for cloning SvS-coding gene	CGMCC
E. coli Trans1T1	*E. coli* host for general cloning	Beijing TransGen Biotech Co., Ltd
E. coli BL21 (DE3)	*E. coli* host for protein expression and fermentation	Beijing TransGen Biotech Co., Ltd

Table S2. Primers used in this study

Primers	Sequence (5'-3')	Purpose
1316-F	TGGTGCCGCGCGGCAGCCATatggccatgaccgtcaacgagatc	Forward primer for cloning SvS-coding gene
1316-R	TCGAGTGCGGCCGCAAGCTTtcaaactccgagcagcgcgtc	Reverse primer for cloning SvS-coding gene
28a-F	AAGCTTGCGGCCGCACTCGA	Forward primer for linearizing pET28a vector
28a-R	ATGGCTGCCGCGCGGCACCA	Reverse primer for linearizing pET28a vector

Table S3. ^{1}H NMR data of spiroviolene in comparison with those of reported

No.	Our Isolated Spiroviolene		Spiroviolene[5,8]
	δ_H, multi (*J* in Hz)^a	δ_H, multi (*J* in Hz)^b	δ_H, multi (*J* in Hz)^b
**1**	4.77, d (2.9)	4.82, d (2.9)	4.81, d (2.9)
**2**	--	--	--
**3**	1.65, m	1.61, m	1.60, m
**4**	1.74, m<br>1.27, m	1.80, m<br>1.39, m	1.79, m<br>1.38, m
**5**	1.74, m<br>1.26, m	1.75, m<br>1.34, m	1.74, m<br>1.33, m
**6**	1.85, m	1.82, m	1.81, m
**7**	--	--	--
**8**	1.93, td (12.8, 7.0)<br>1.68, m	1.93, td (12.8, 7.0)<br>1.70, m	1.92, ddd (12.7, 6.9, 6.9)<br>1.69, m
**9**	1.68, m<br>1.02, m	1.73, m<br>1.09, m	1.72, m<br>1.09, dddd (12.2, 12.2, 11.3, 7.6)
**10**	2.68, dtd<br>(12.5, 6.4, 2.9)	2.77, dtd<br>(12.6, 6.4, 2.9)	2.77, dddd<br>(12.5, 6.4, 6.4, 2.9)
**11**	--	--	--
**12**	1.67, m<br>1.55, m	1.74, m<br>1.59, m	1.73, m<br>1.59, m
**13**	1.55, m<br>1.39, m	1.68, m<br>1.44, m	1.67, m<br>1.43, dddd (11.8, 6.6, 1.5, 1.5)
**14**	1.53, m	1.59, m	1.58, m
**15**	--	--	--
**16**	1.02, s	1.05, s	1.04, s
**17**	0.99, s	1.04, s	1.03, s
**18**	1.29, s	1.34, s	1.34, s
**19**	0.88, d (6.7)	0.98, d (6.8)	0.97, d (6.7)
**20**	0.86, d (6.6)	0.95, d (6.8)	0.94, d (6.7)

^{a}recorded in $CDCl_3$ (400 MHz); ^{b}recorded in C_6D_6 (700 MHz).

Table S4. ^{13}C NMR data of spiroviolene in comparison with those of reported

No.	Our Isolated Spiroviolene		Spiroviolene[5,8]
	δ_C, type[a]	δ_C, type[b]	δ_C, type[b]
**1**	128.7, CH	129.0, CH	128.9, CH
**2**	148.7, qC	148.9, qC	148.9, qC
**3**	44.5, CH	44.7, CH	44.7, CH
**4**	31.0, CH_2	31.3, CH_2	31.3, CH_2
**5**	30.4, CH_2	30.7, CH_2	30.7, CH_2
**6**	46.4, CH	46.6, CH	46.6, CH
**7**	53.5, qC	53.8, qC	53.8, qC
**8**	39.3, CH_2	39.6, CH_2	39.5, CH_2
**9**	32.8, CH_2	33.1, CH_2	33.1, CH_2
**10**	59.1, CH	59.4, CH	59.4, CH
**11**	63.5, qC	63.7, qC	63.7, qC
**12**	38.4, CH_2	38.6, CH_2	38.6, CH_2
**13**	40.5, CH_2	40.8, CH_2	40.8, CH_2
**14**	65.9, CH	66.1, CH	66.0, CH
**15**	41.2, qC	41.3, qC	41.3, qC
**16**	29.1, CH_3	29.2, CH_3	29.1, CH_3
**17**	26.0, CH_3	26.1, CH_3	26.1, CH_3
**18**	32.3, CH_3	32.4, CH_3	32.4, CH_3
**19**	15.0, CH_3	15.2, CH_3	15.2, CH_3
**20**	15.0, CH_3	15.1, CH_3	15.1, CH_3

[a]recorded in $CDCl_3$ (100 MHz); [b]recorded in C_6D_6 (150 MHz).

Table S5. ^{1}H NMR data of 1α-hydroxyspiroviolane (**9**), 9α-hydroxyspiroviolane (**10**) and 9β-hydroxy-spiroviolane (**11**).

**No.**	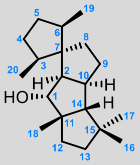<br>**9**	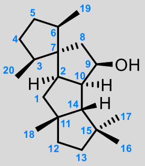<br>**11**	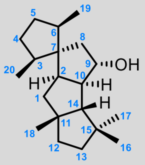<br>**10**
	**δ_H, multi (*J* in Hz)[a]**	**δ_H, multi (*J* in Hz)[a]**	**δ_H, multi (*J* in Hz)[a]**
**1**	3.74, d (9.6)	1.74, m<br>1.34, m	1.71, m<br>1.26, m
**2**	2.01, m	2.36, dt (11.8, 7.2)	2.37, dt (12.4, 7.1)
**3**	1.62, m	1.58, m	2.02, m
**4**	2.00, m<br>1.41, m	2.03, m<br>1.36, m	2.05, m<br>1.39, m
**5**	1.71, m<br>1.41, m	1.71, m<br>1.36, m	1.67, m<br>1.33, m
**6**	1.93, m	1.75, m	1.67, m
**7**	--	--	--
**8**	1.54, m<br>1.40, m	2.05, m<br>1.46, m	2.00, m<br>1.50, m
**9**	1.94, m<br>1.20, m	4.20, dt (8.9, 7.1)	4.05, ddd (8.8, 5.9, 2.8)
**10**	2.16, m	2.27, ddd (8.8, 5.0, 3.5)	2.12, t (6.2)
**11**	--	--	--
**12**	1.94, m<br>1.20, m	1.59, m	1.55, t (6.9)
**13**	1.49, m<br>1.42, m	1.50, m<br>1.46, m	1.43, m
**14**	1.40, m	1.78, m	1.68, m
**15**	--	--	--
**16**	1.04, s	1.05, s	1.04, s
**17**	0.97, s	0.96, s	0.96, s
**18**	1.24, s	1.19, s	1.20, s
**19**	1.12, s	0.93, s	1.01, s
**20**	0.95, s	0.95, s	0.91, s

[a]recorded in $CDCl_3$ (600 MHz).

Table S6. ^{13}C NMR data of 1α-hydroxy-spiroviolane (**9**), 9α-hydroxy-spiroviolane (**10**) and 9β-hydroxy-spiroviolane (**11**).

**No.**	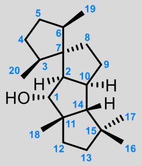 **9**	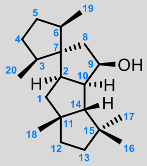 **11**	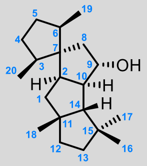 **10**
	**δ_C, type[a]**	**δ_C, type[a]**	**δ_C, type[a]**
**1**	82.8, CH	45.5, CH_2	45.0, CH_2
**2**	53.5, CH	48.3, CH	48.8, CH
**3**	41.9, CH	42.0, CH	42.6, CH
**4**	30.9, CH_2	31.9, CH_2	31.6, CH_2
**5**	32.5, CH_2	32.3, CH_2	32.2, CH_2
**6**	42.8, CH	42.7, CH	43.4, CH
**7**	59.1, qC	55.4, qC	58.0, qC
**8**	38.4, CH_2	50.0, CH_2	49.6, CH_2
**9**	32.3, CH_2	72.9, CH	81.5, CH
**10**	42.2, CH	50.7, CH	58.5, CH
**11**	56.0, qC	52.8, qC	52.8, qC
**12**	32.3, CH_2	40.7, CH_2	40.9, CH_2
**13**	41.4, CH_2	42.1, CH_2	41.9, CH_2
**14**	66.3, CH	60.0, CH	66.4, CH
**15**	42.9, qC	42.7, qC	42.7, qC
**16**	31.1, CH_3	31.3, CH_3	31.3, CH_3
**17**	25.7, CH_3	26.1, CH_3	26.0, CH_3
**18**	30.5, CH_3	29.3, CH_3	32.0, CH_3
**19**	18.5, CH_3	18.5, CH_3	19.6, CH_3
**20**	19.5, CH_3	20.3, CH_3	18.4, CH_3

[a]recorded in $CDCl_3$ (150 MHz).

Figure S1. Key ^1H-^1H COSY, HMBC and NOESY correlations of spiroviolene **(1)**, 1α-hydroxy-spiroviolane (**9**), 9α-hydroxyspiroviolane (**10**) and 9β-hydroxyspiroviolane (**11**).

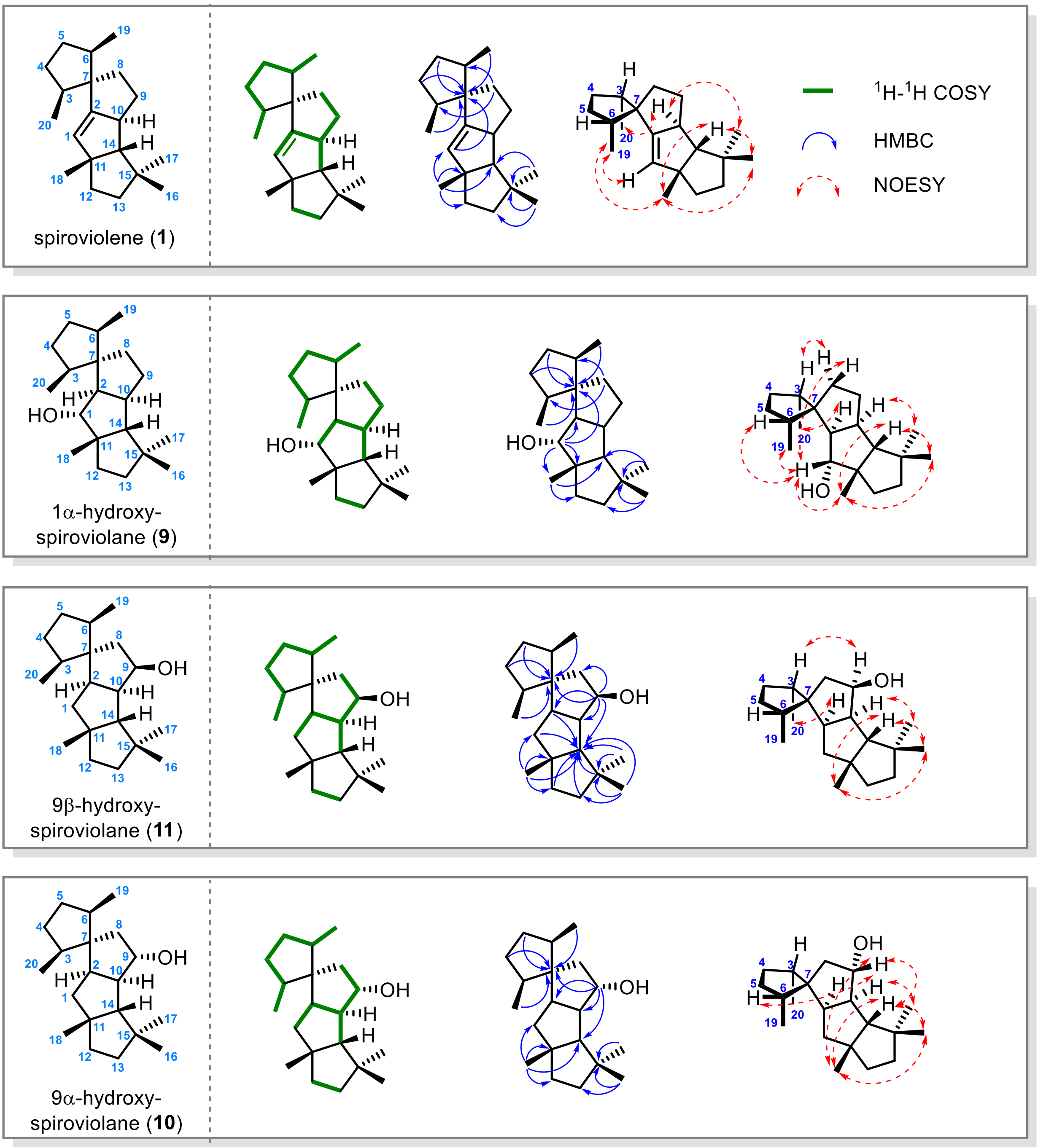

Figure 2a. ^{1}H NMR spectrum of spiroviolene 1 (600 MHz, C_6D_6)

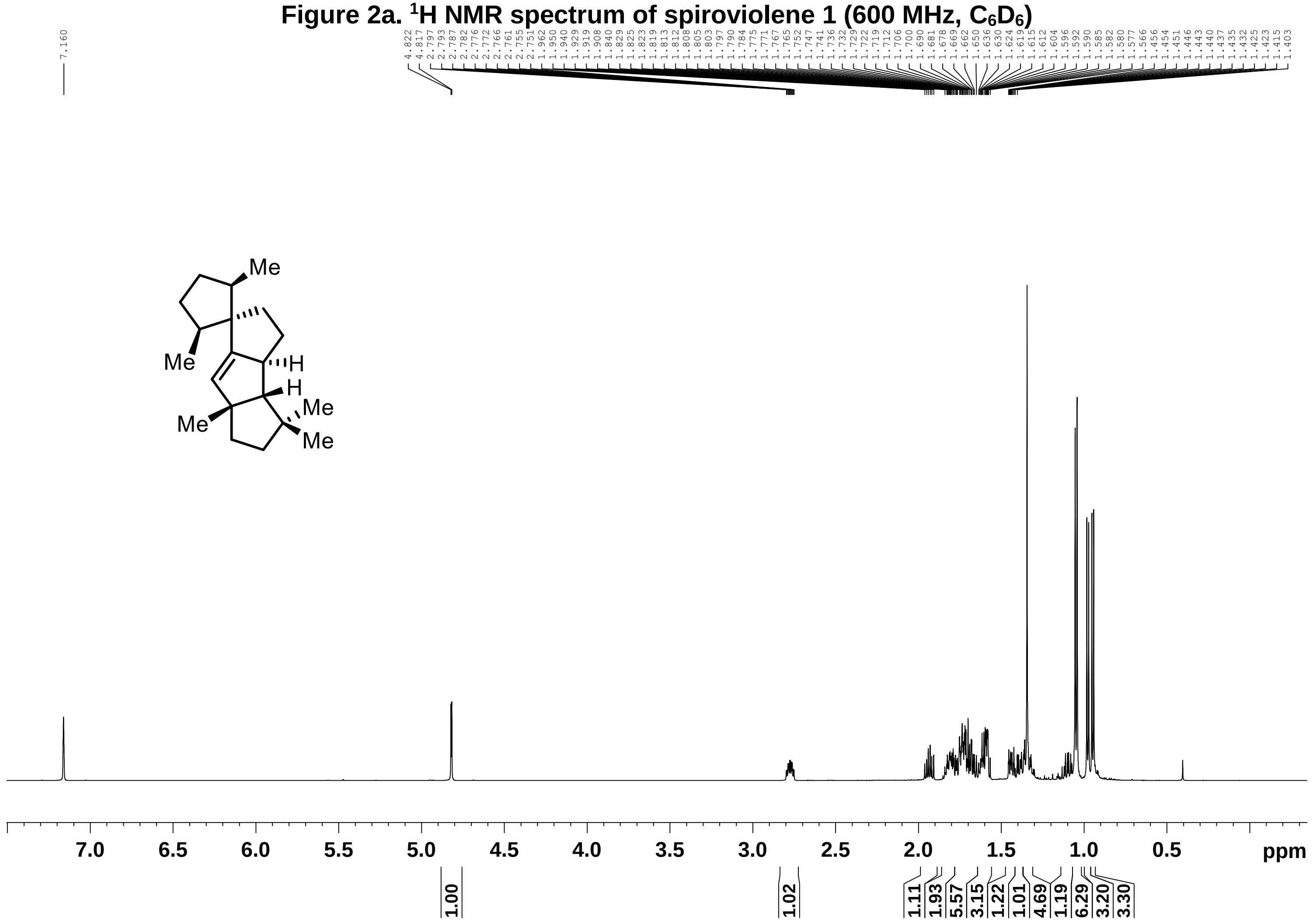

Figure 2b. ^{1}H NMR spectrum of spiroviolene 1 (600 MHz, C_6D_6)

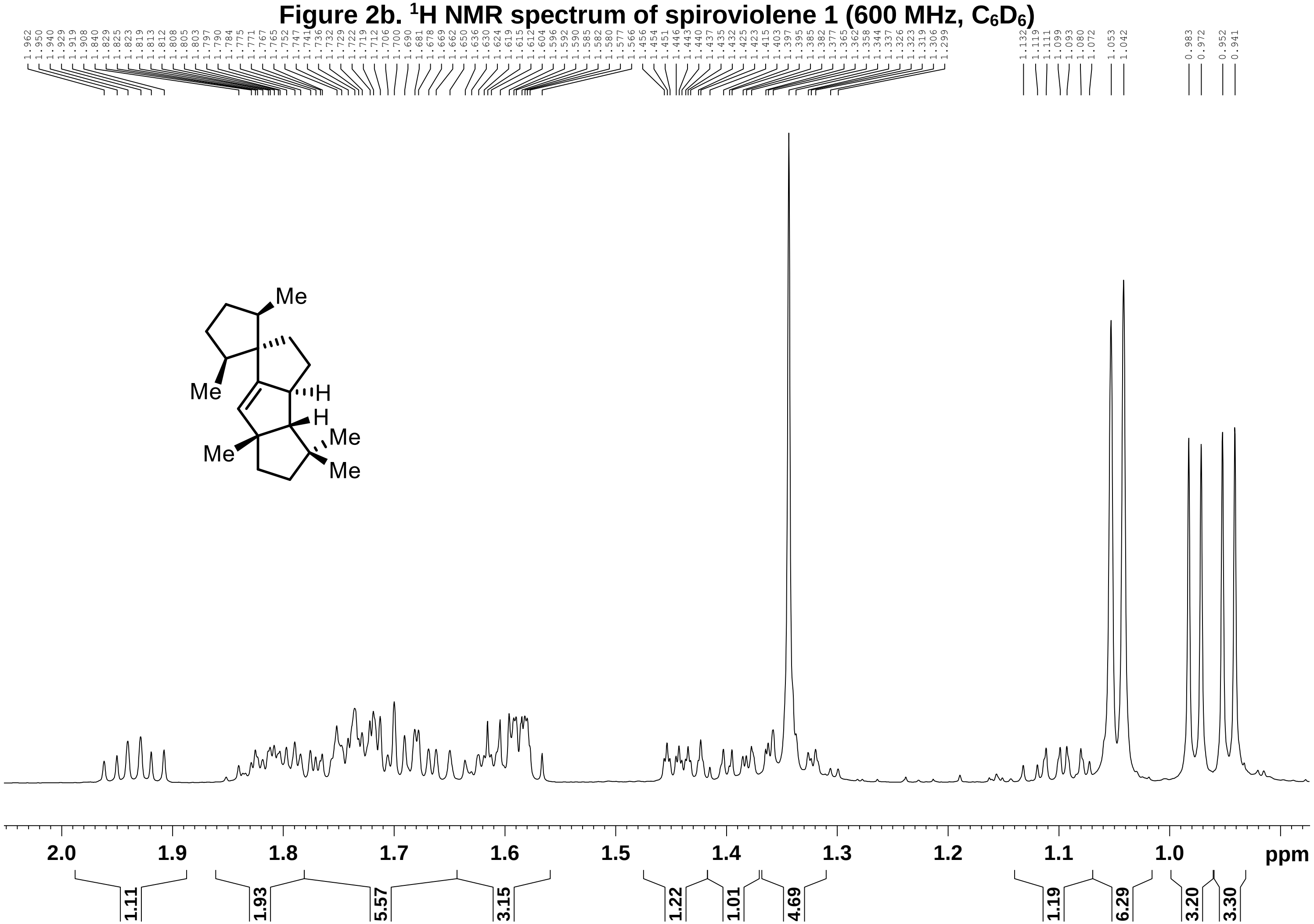

Figure 2c. ^{13}C NMR spectrum of spiroviolene 1 (150 MHz, C_6D_6)

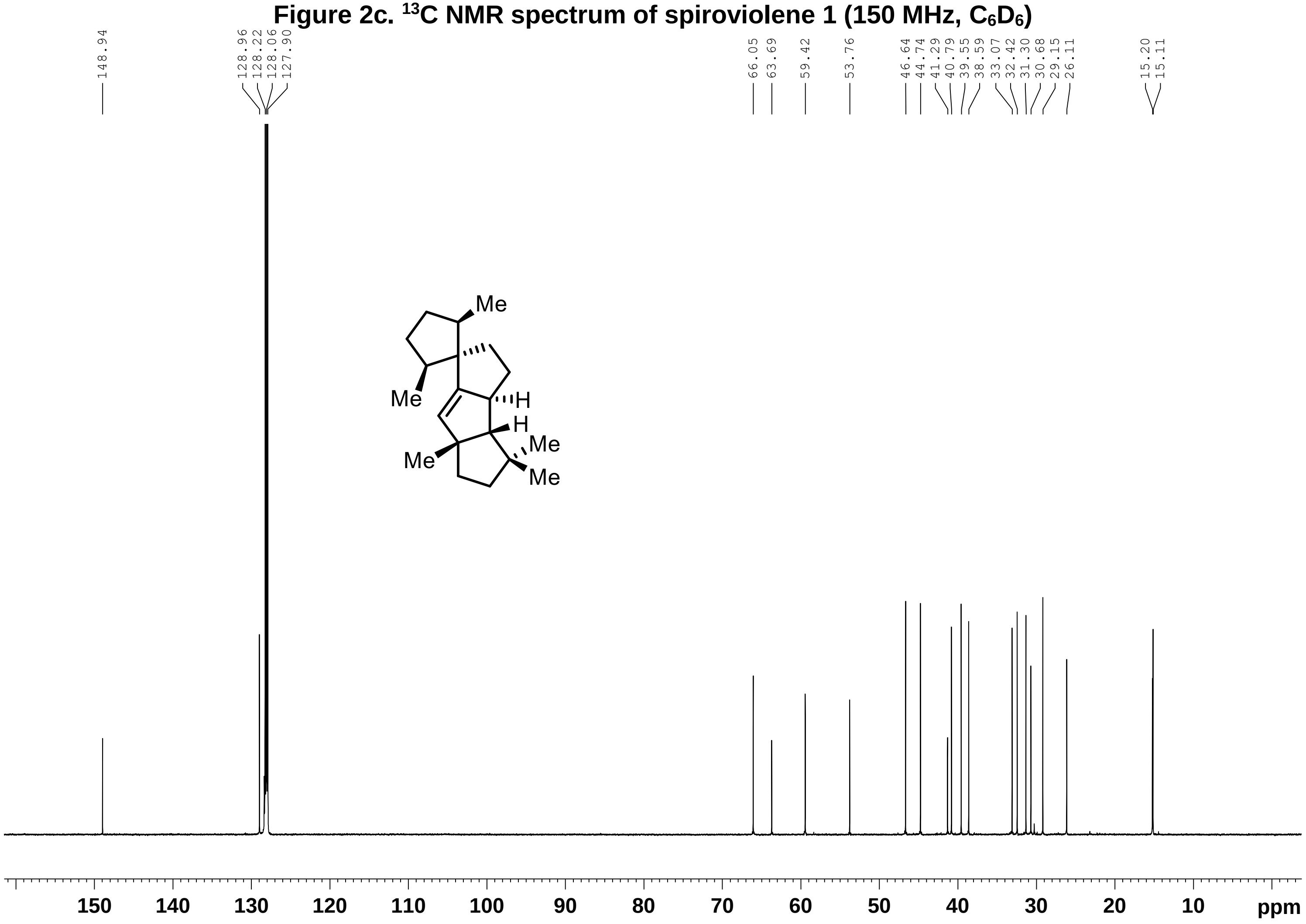

Figure 2d. ^1H-^1H COSY spectrum of spiroviolene 1 (C_6D_6)

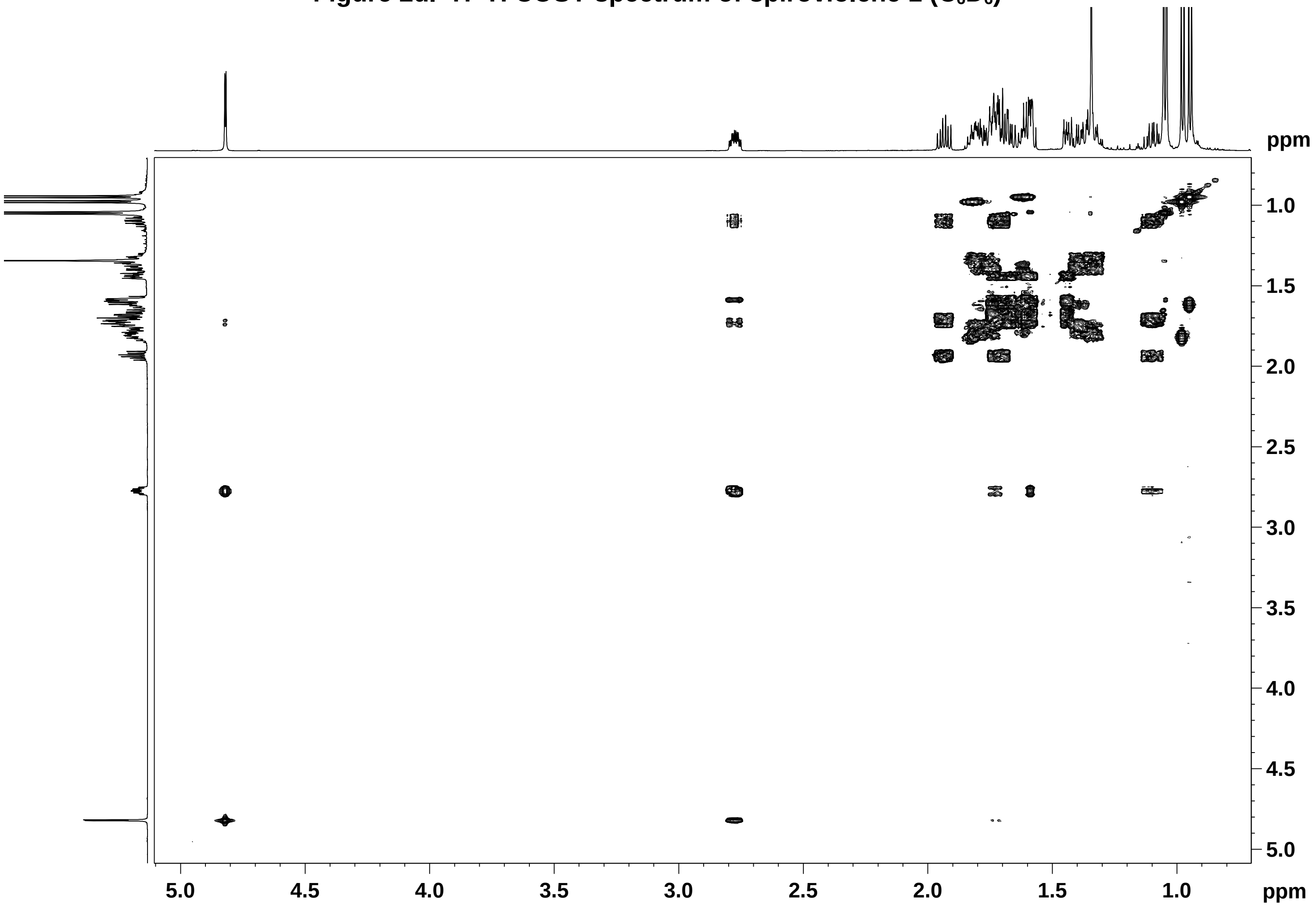

Figure 2e. HSQC spectrum of spiroviolene 1 (C_6D_6)

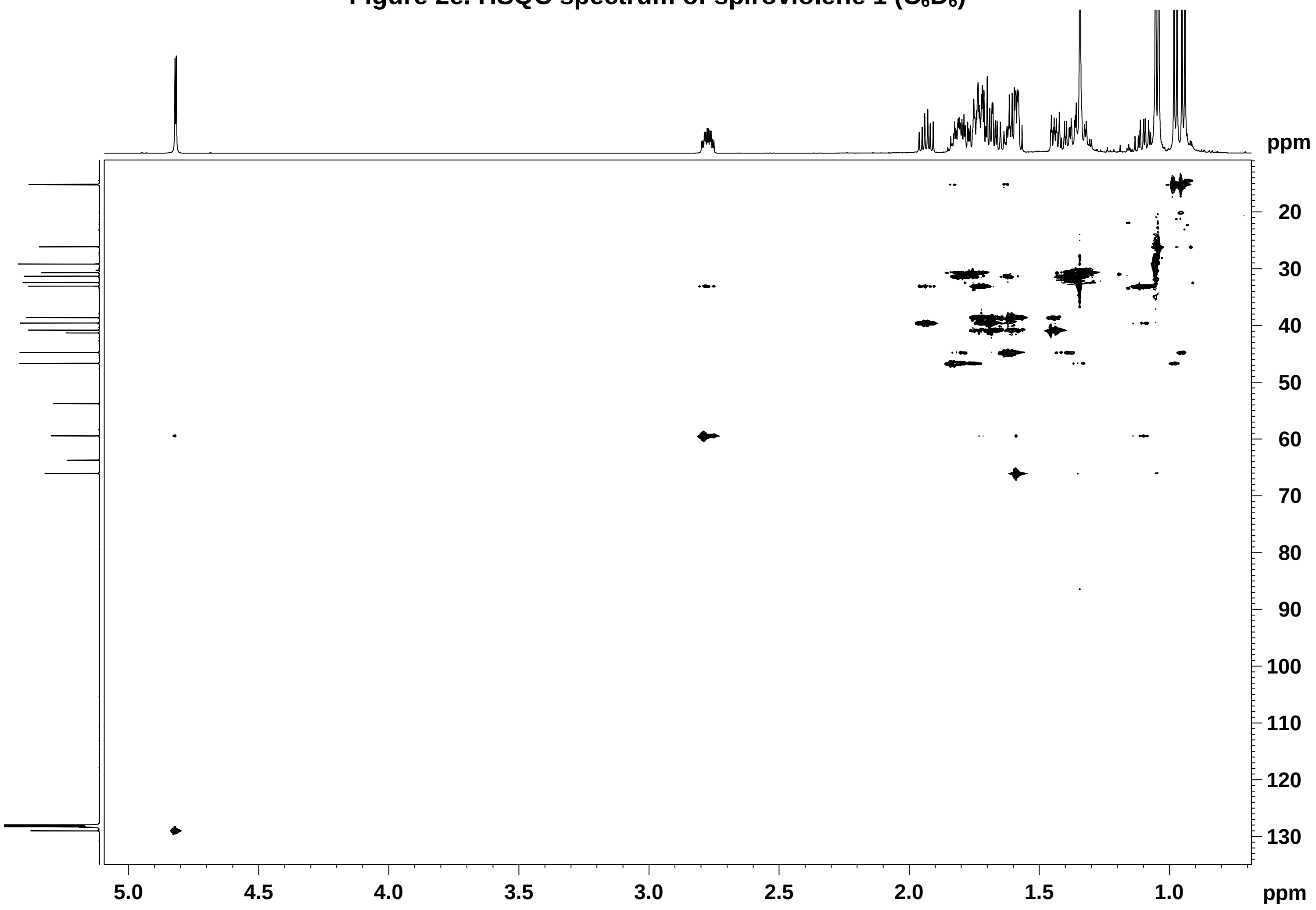

Figure 2f. HMBC spectrum of spiroviolene 1 (C_6D_6)

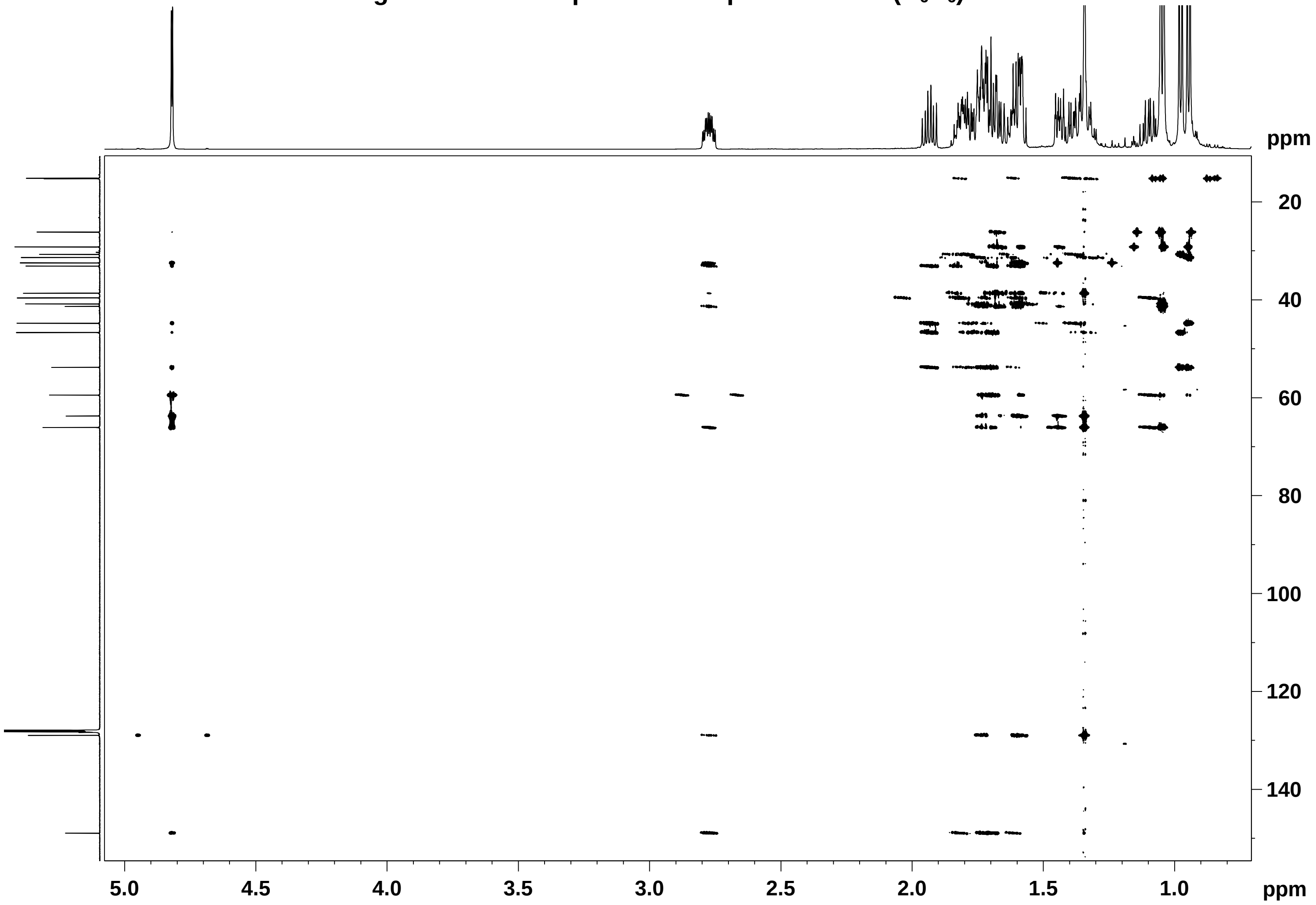

Figure 2g. NOESY spectrum of spiroviolene 1 (C_6D_6)

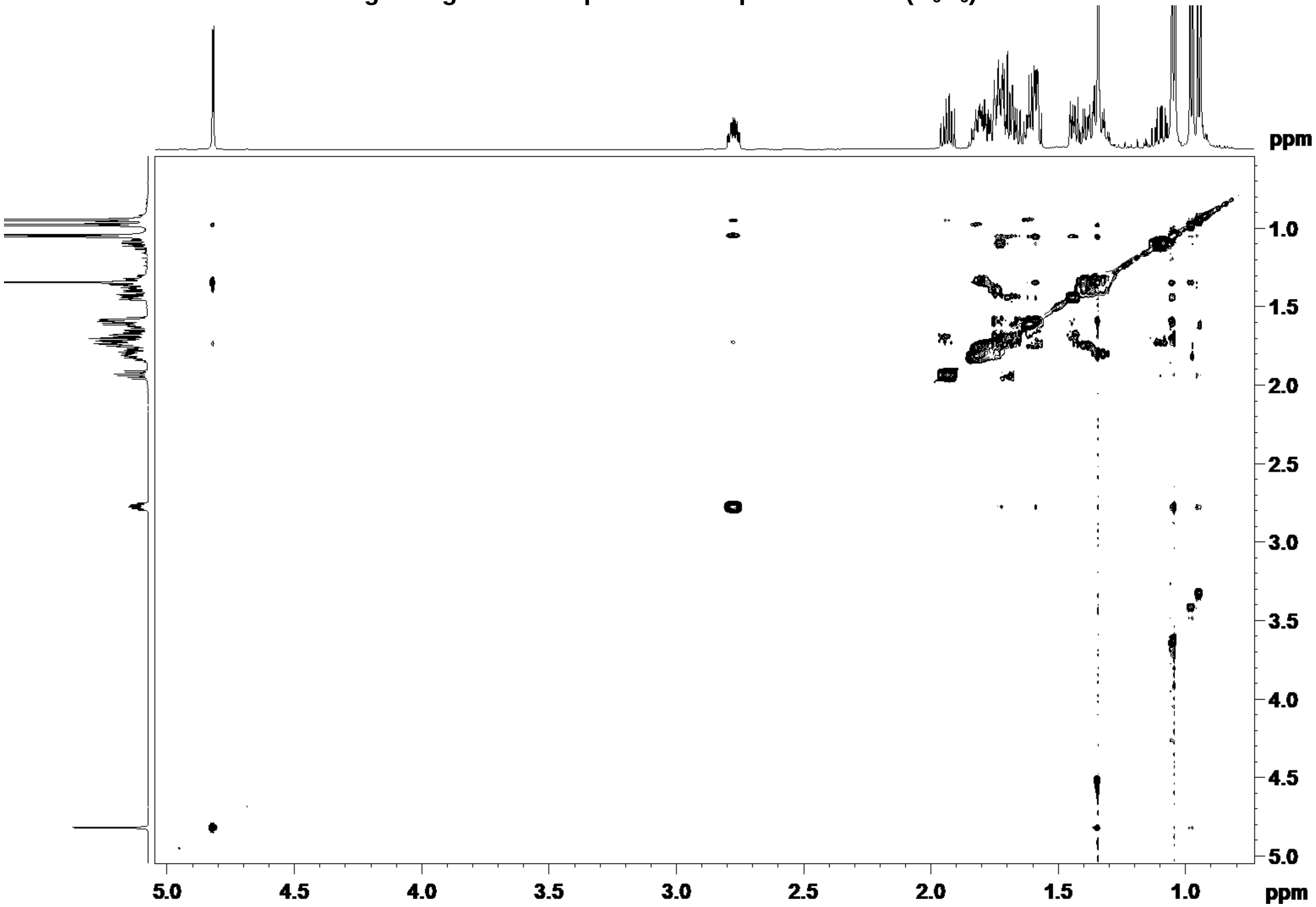

Figure 3a. ^{1}H NMR spectrum of spiroviolene 1 (400 MHz, $CDCl_3$)

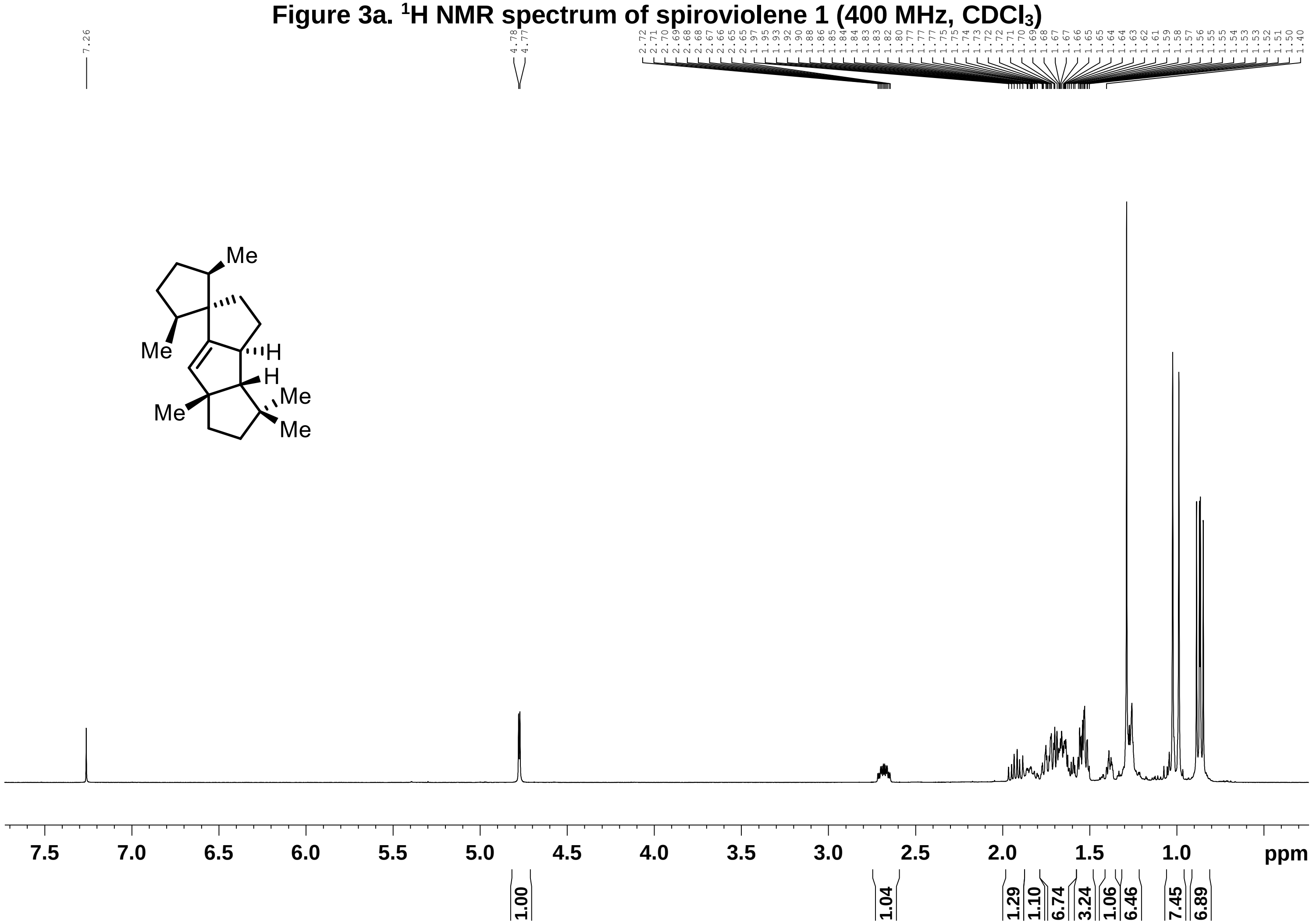

Figure 3b. ^{1}H NMR spectrum of spiroviolene 1 (400 MHz, $CDCl_3$)

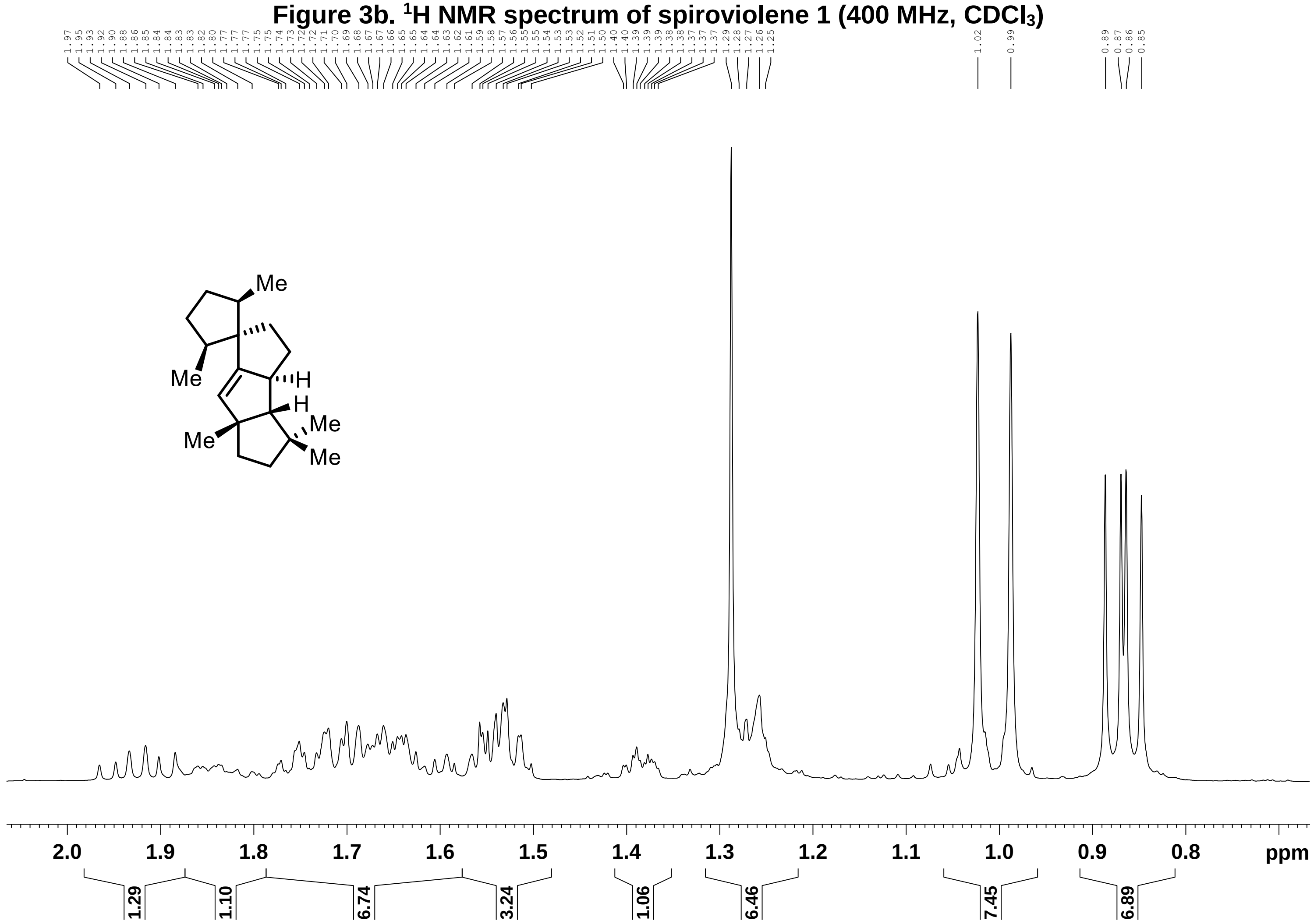

Figure 3c. ^{13}C NMR spectrum of spiroviolene 1 (100 MHz, $CDCl_3$)

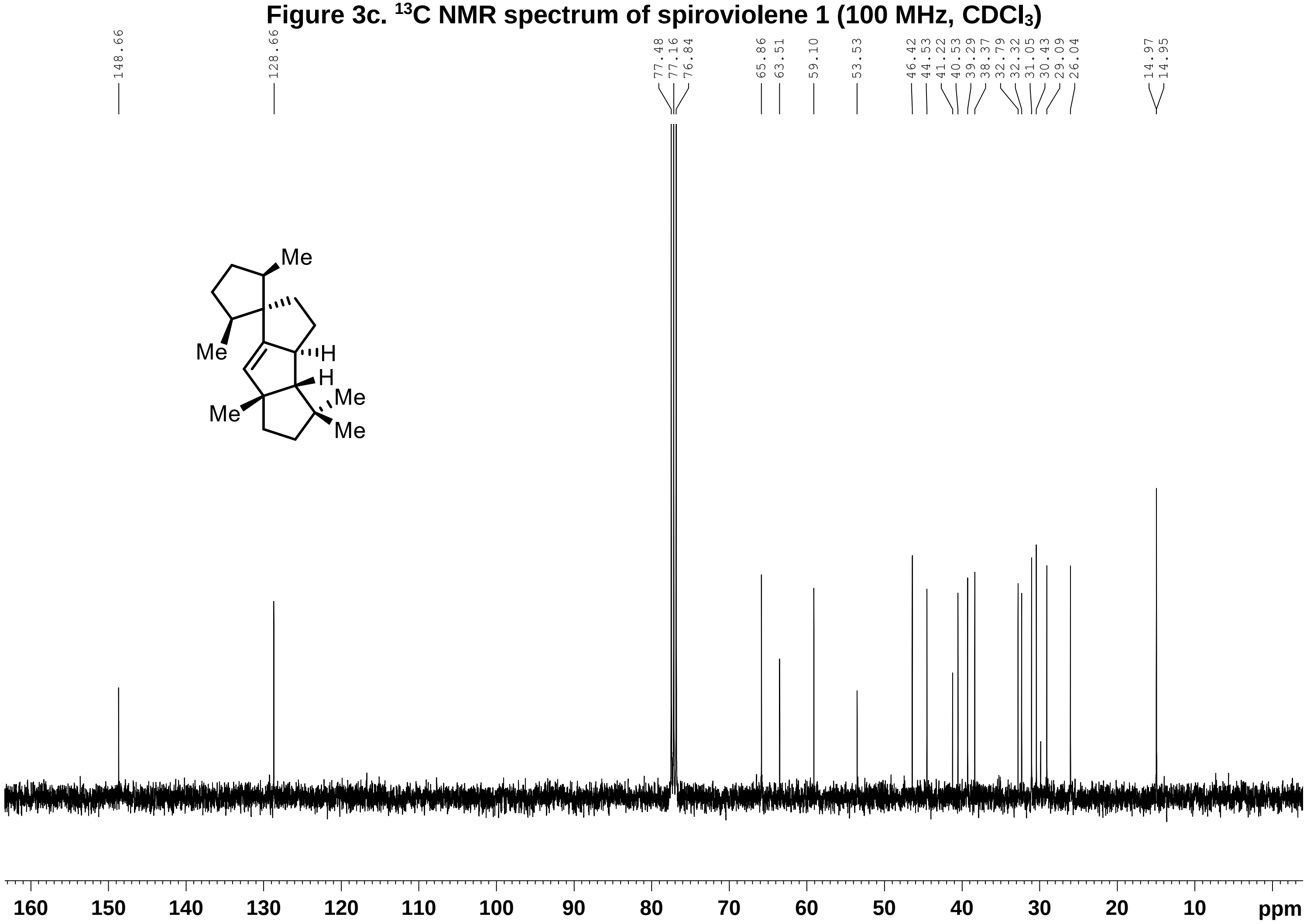

Figure 3d. ^1H-^1H **COSY spectrum of spiroviolene 1 ($CDCl_3$)**

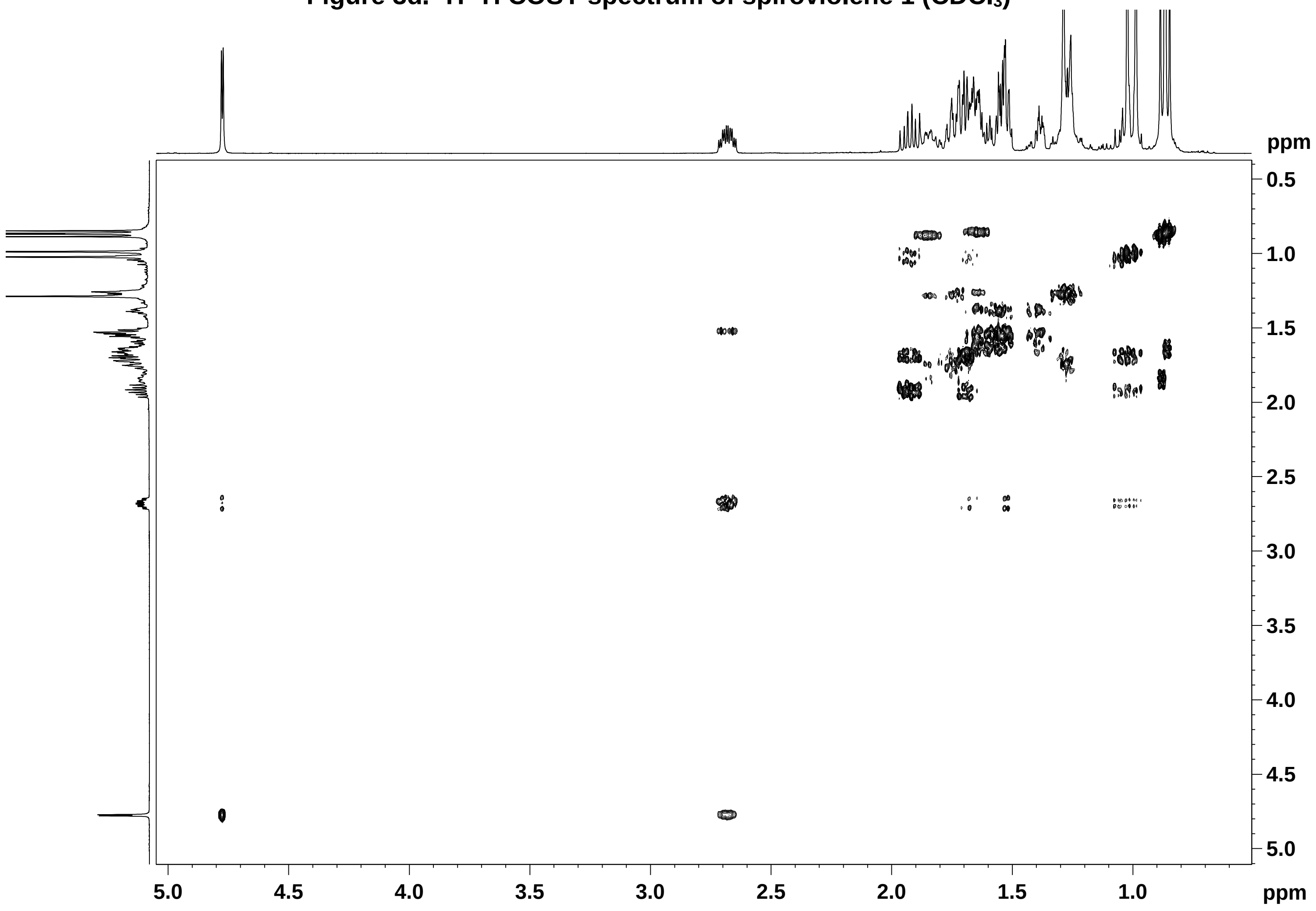

Figure 3e. HSQC spectrum of spiroviolene 1 ($CDCl_3$)

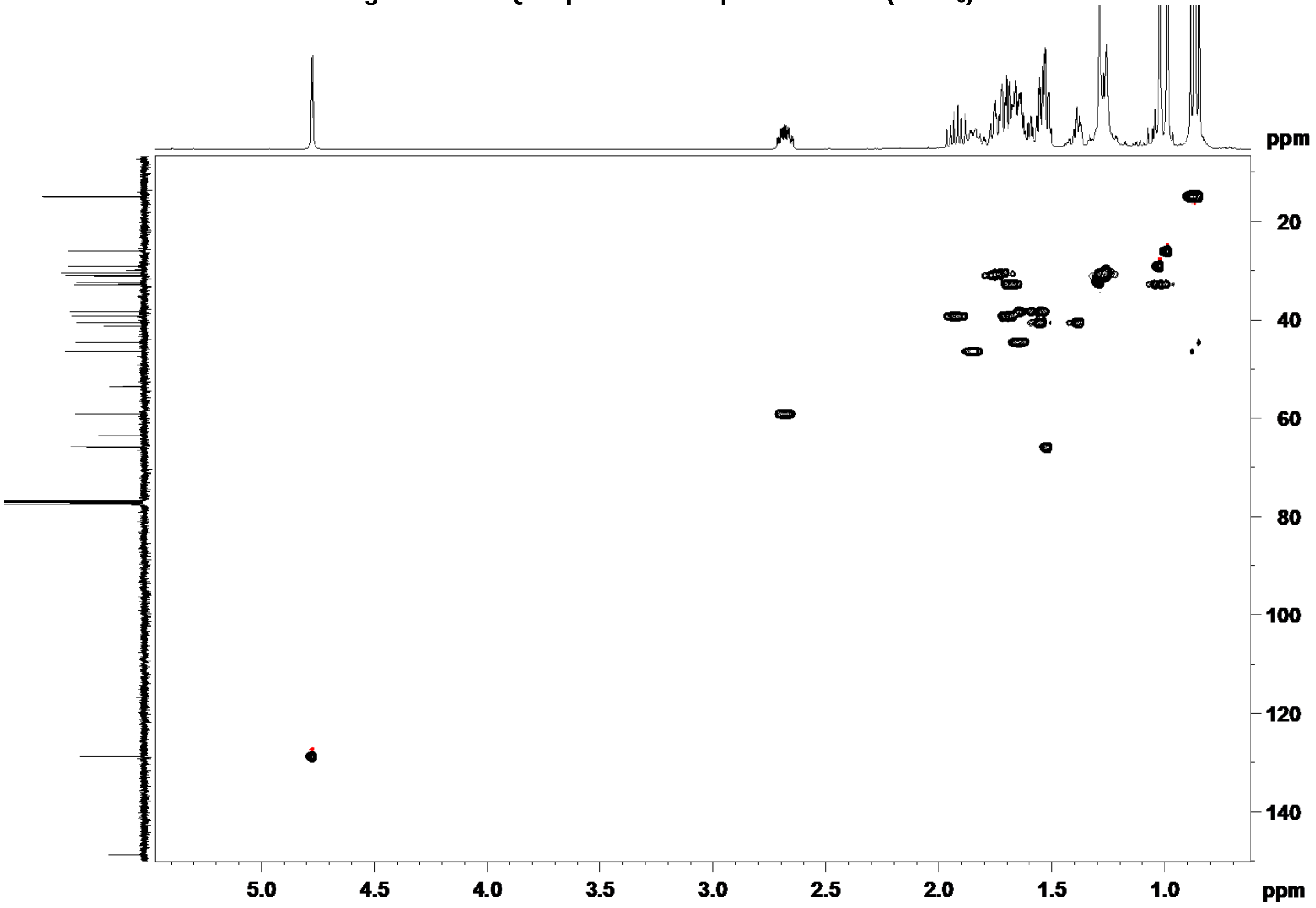

Figure 3f. HMBC spectrum of spiroviolene 1 ($CDCl_3$)

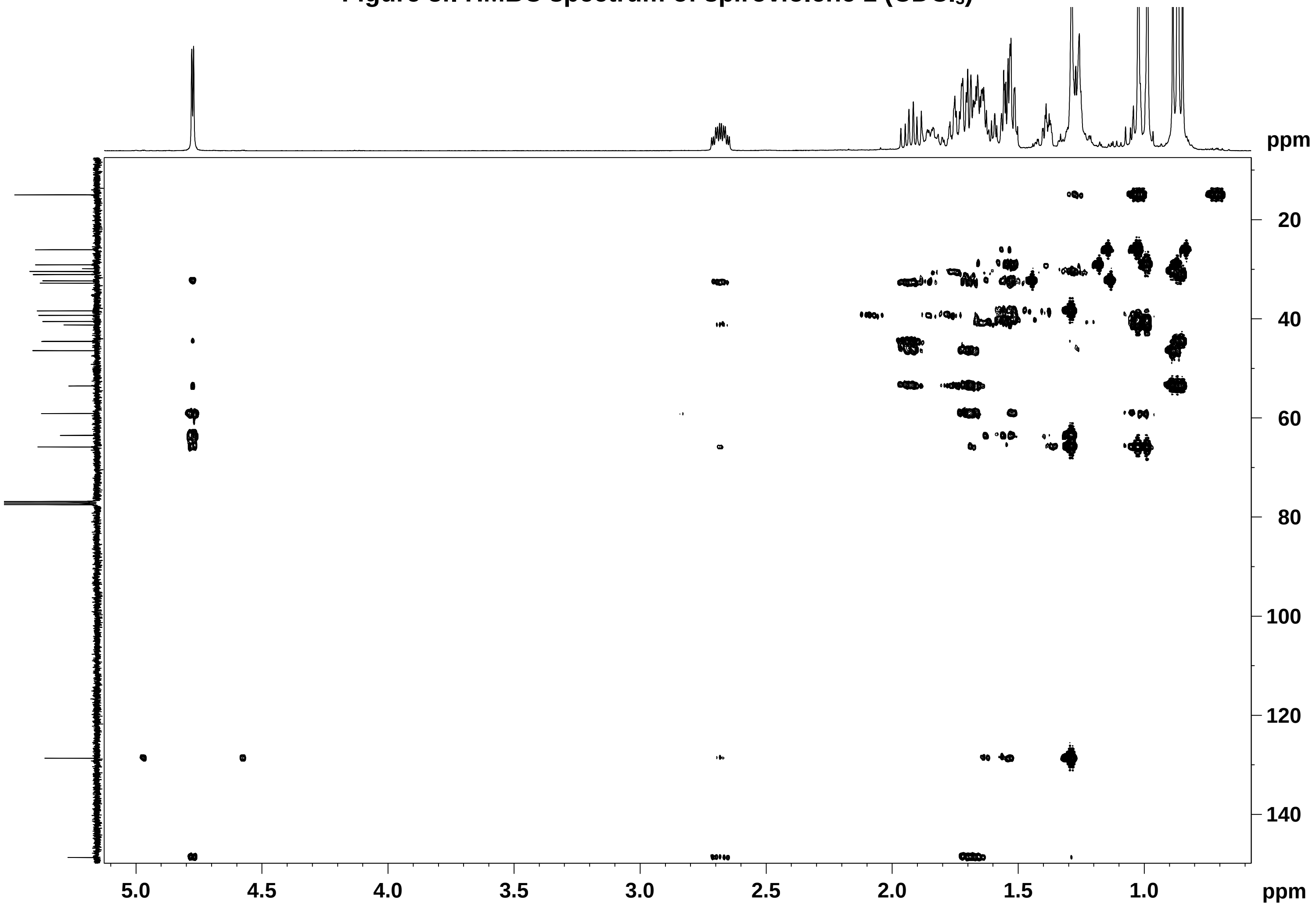

Figure 3g. NOESY spectrum of spiroviolene 1 ($CDCl_3$)

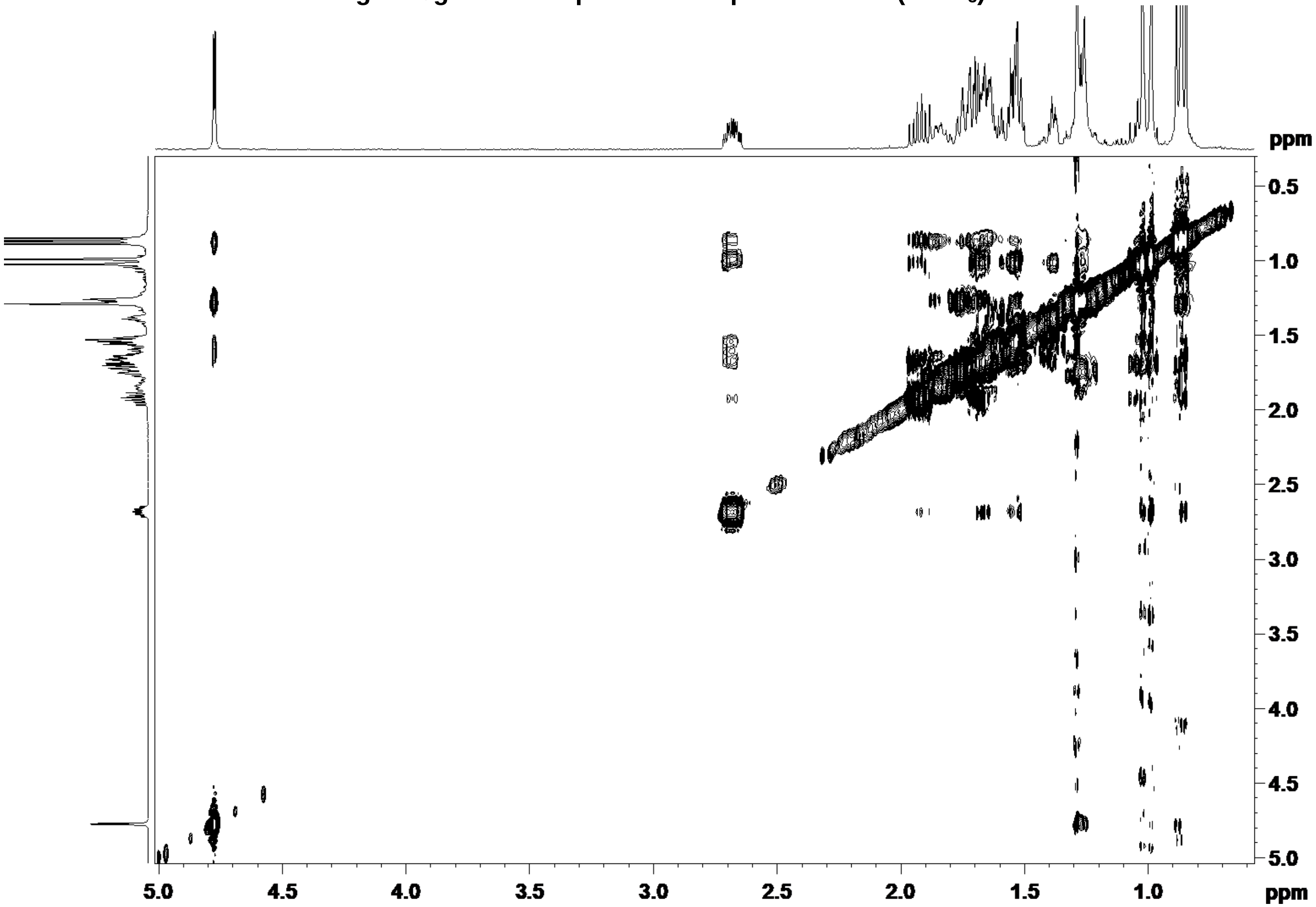

Figure 4a. ^{1}H NMR spectrum of 1α-hydroxy-spiroviolane 9 (600 MHz, $CDCl_3$)

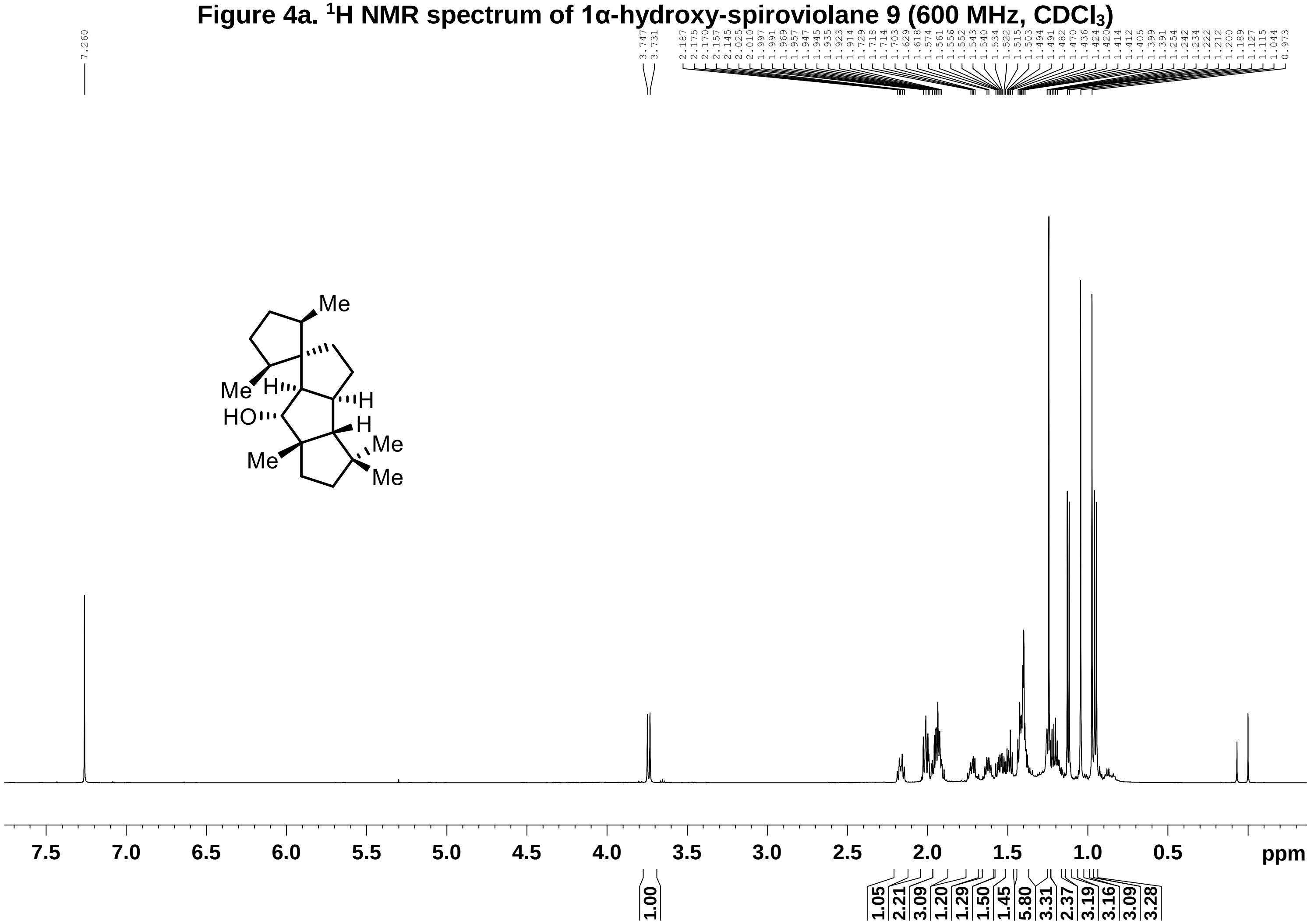

Figure 4b. ¹H NMR spectrum of 1α-hydroxy-spiroviolane 9 (600 MHz, $CDCl_3$)

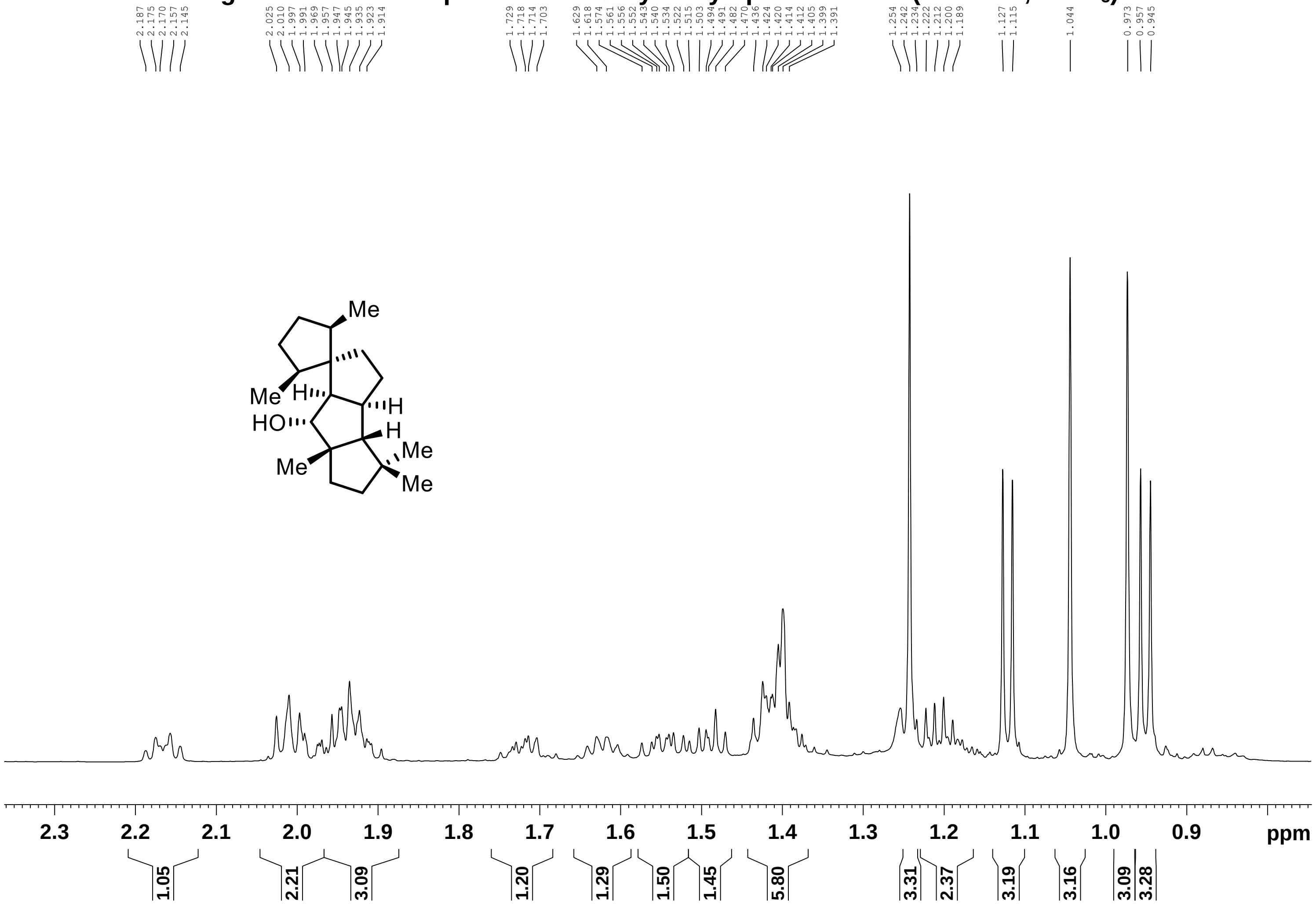

Figure 4c. ^{13}C NMR spectrum of 1α-hydroxy-spiroviolane 9 (150 MHz, $CDCl_3$)

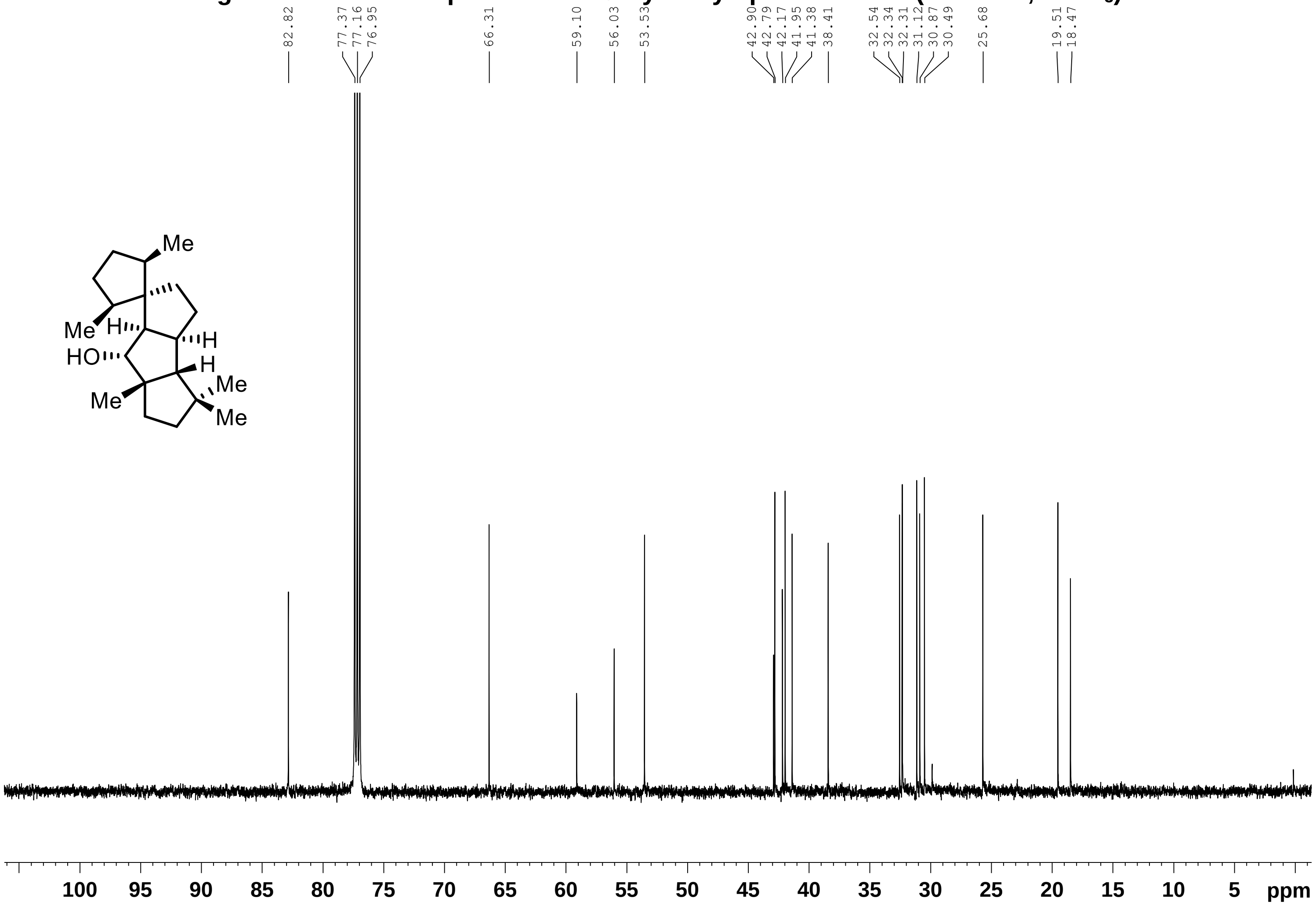

Figure 4d. ^{1}H-^{1}H COSY spectrum of 1α-hydroxy-spiroviolane 9 ($CDCl_3$)

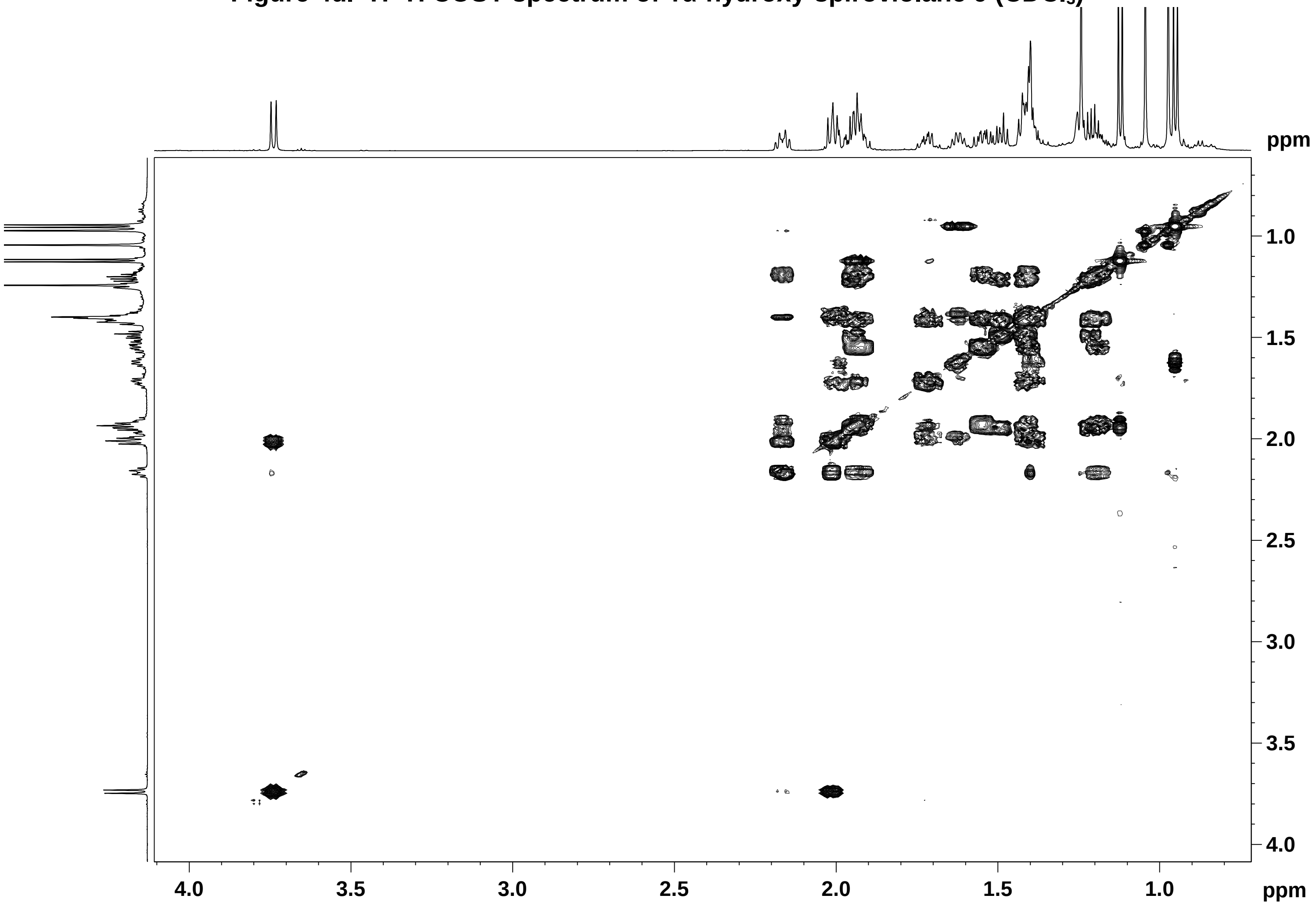

Figure 4e. HSQC spectrum of 1α-hydroxy-spiroviolane 9 ($CDCl_3$)

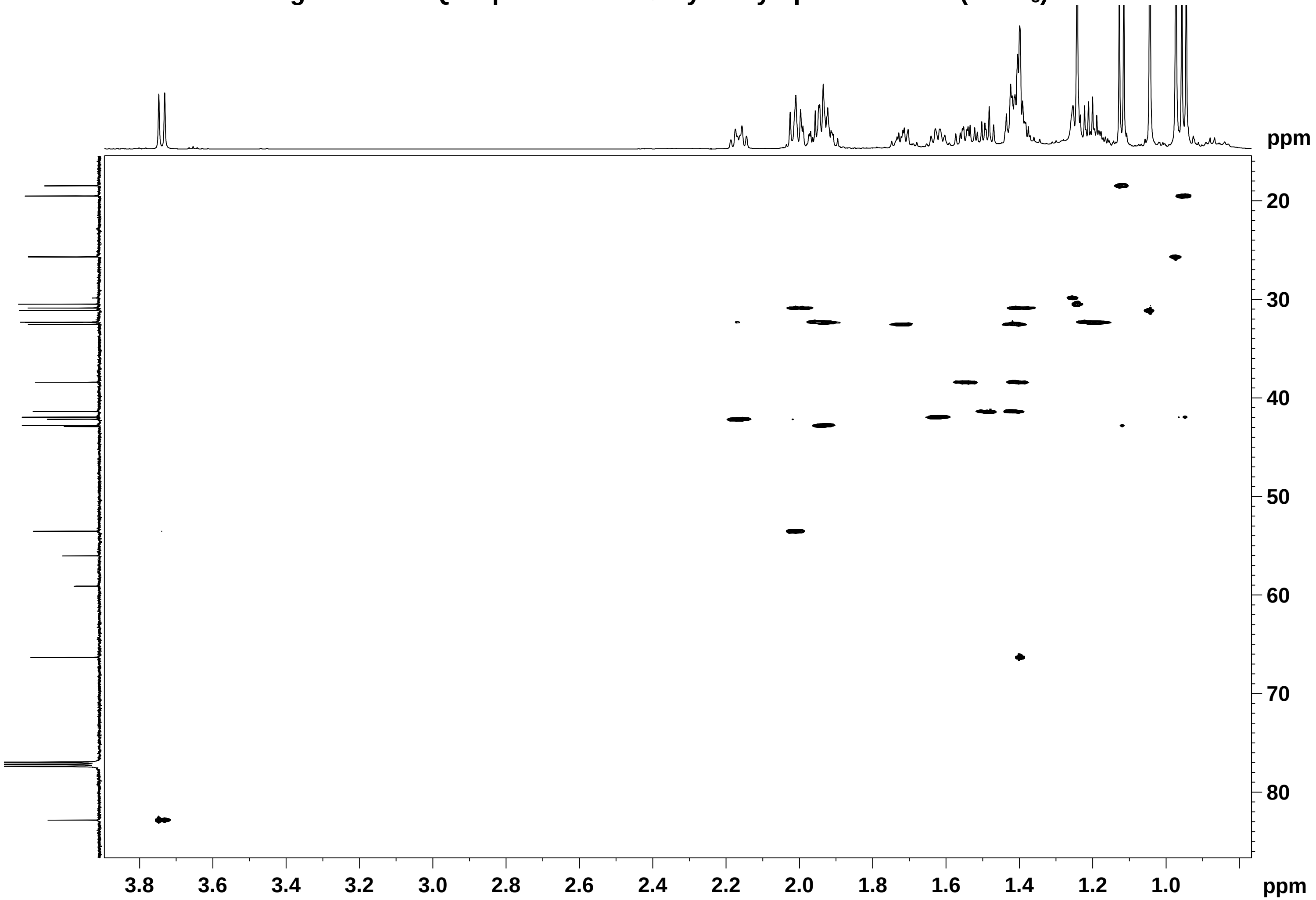

Figure 4f. HMBC spectrum of 1α-hydroxy-spiroviolane 9 ($CDCl_3$)

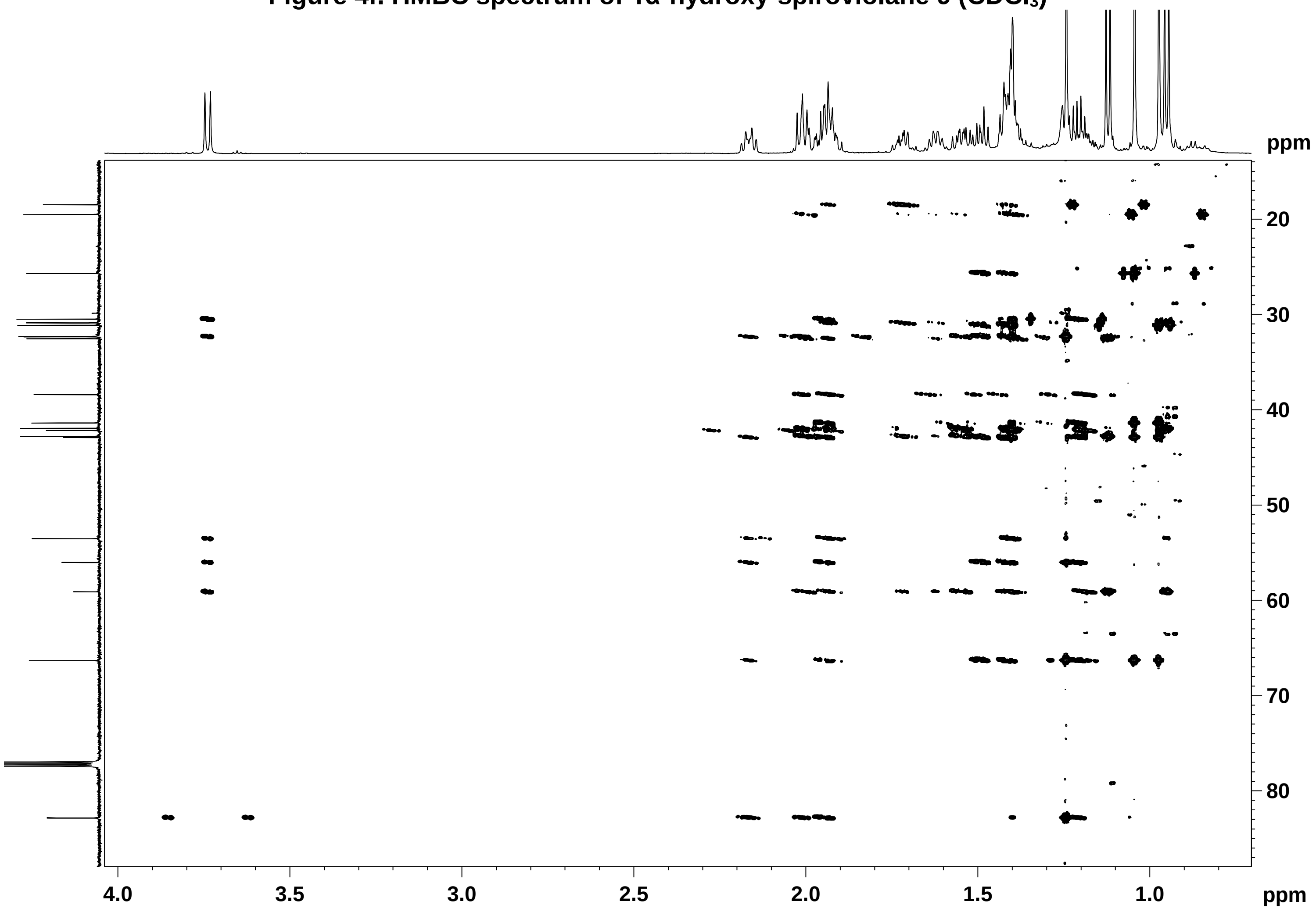

Figure 4g. NOESY spectrum of 1α-hydroxy-spiroviolane 9 ($CDCl_3$)

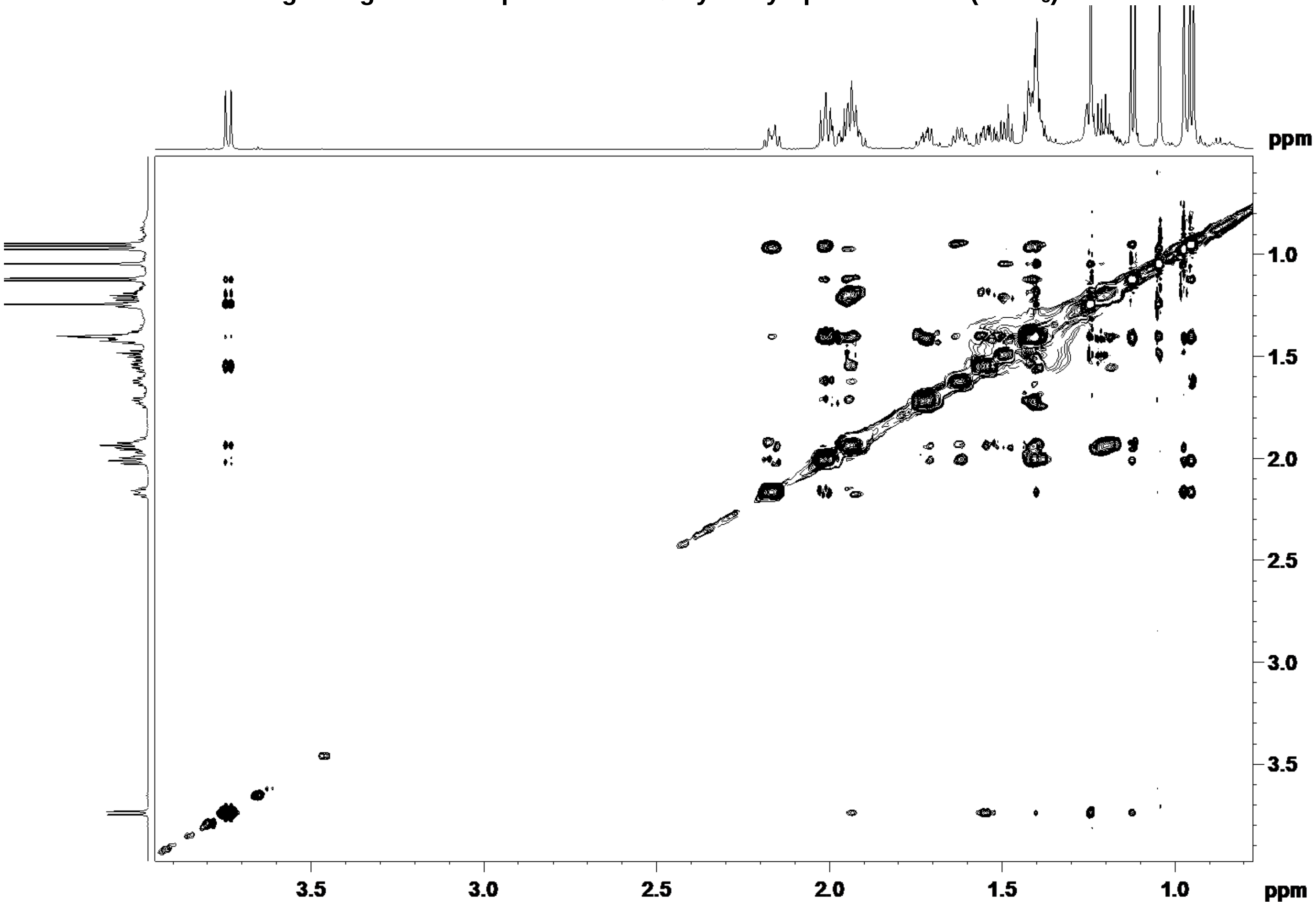

Figure 5a. ¹H NMR spectrum of compound 9β-hydroxy-spiroviolane 11 (600 MHz, $CDCl_3$)

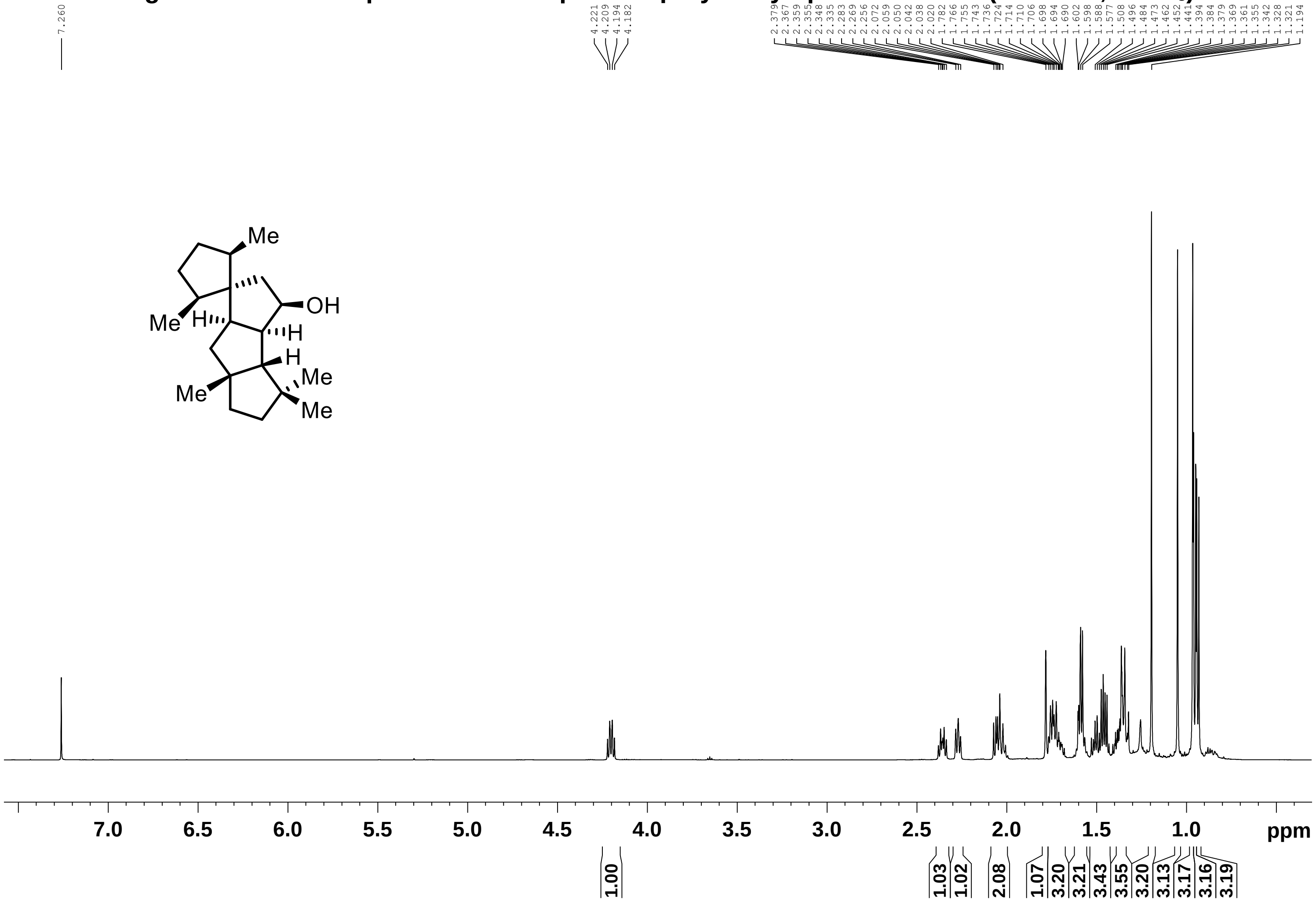

Figure 5b. ^{1}H NMR spectrum of 9β-hydroxy-spiroviolane 11 (600 MHz, $CDCl_3$)

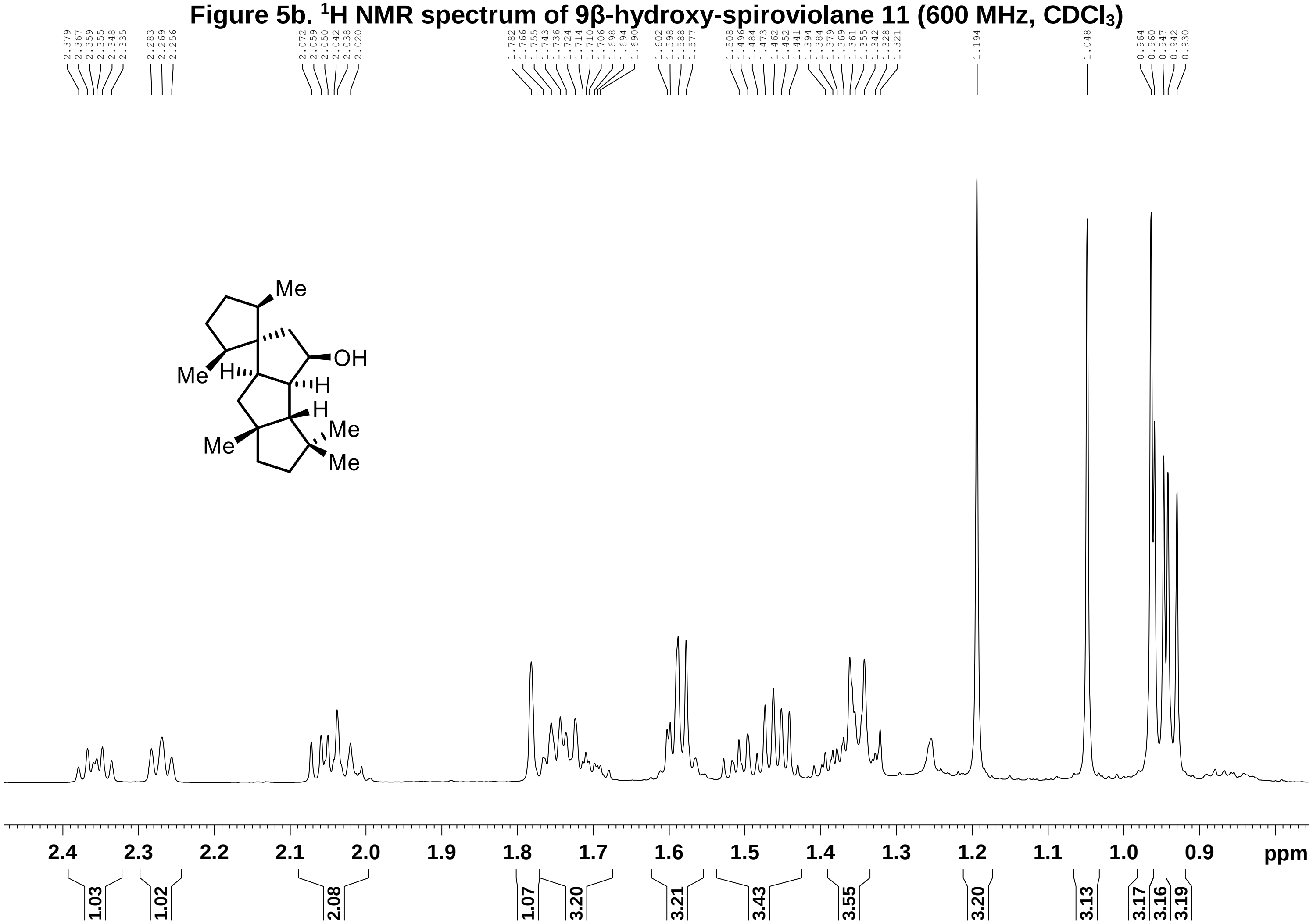

Figure 5c. ^{13}C NMR spectrum of 9β-hydroxy-spiroviolane 11 (150 MHz, $CDCl_3$)

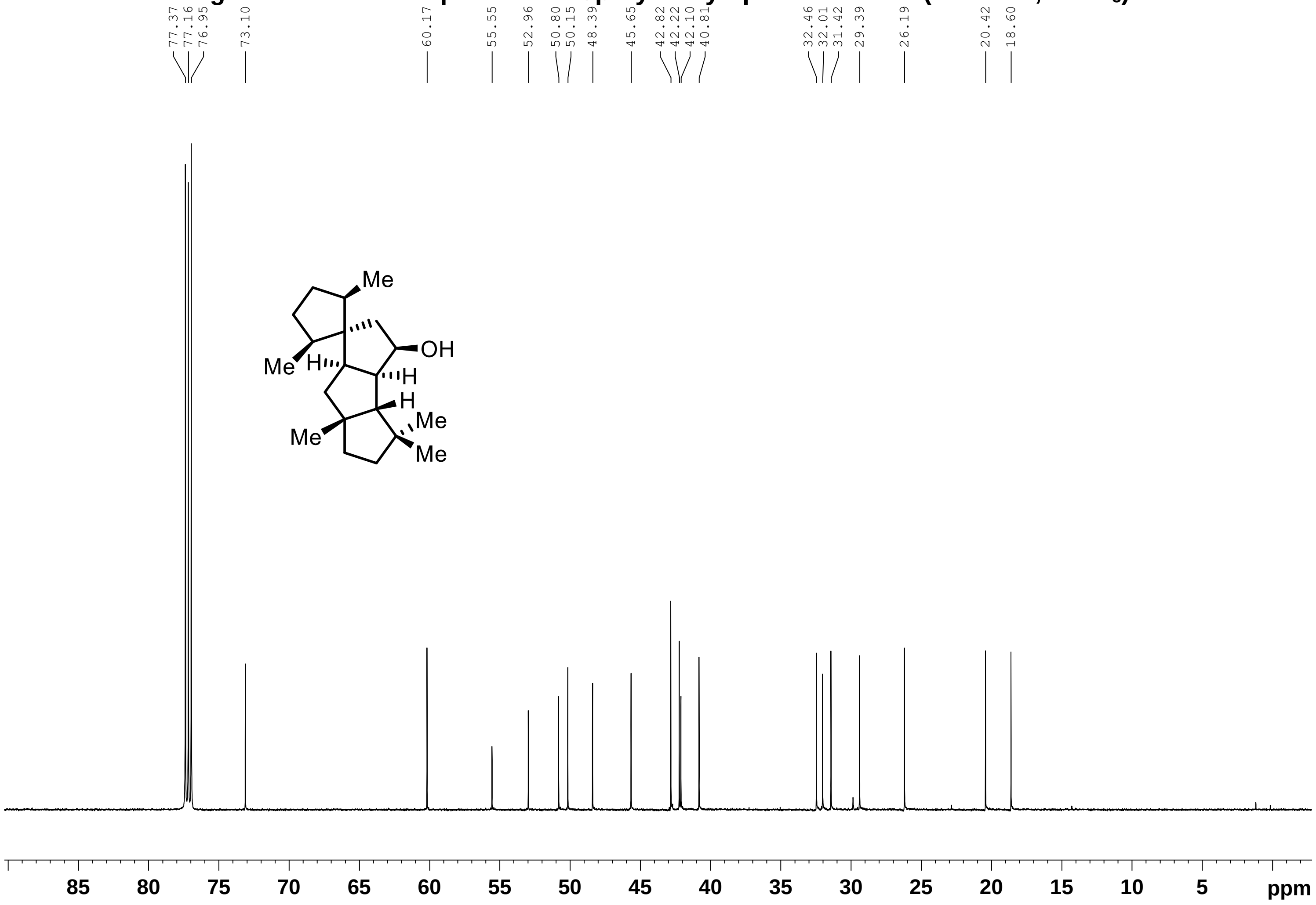

Figure 5d. ^{1}H-^{1}H COSY spectrum of 9β-hydroxy-spiroviolane 11 ($CDCl_3$)

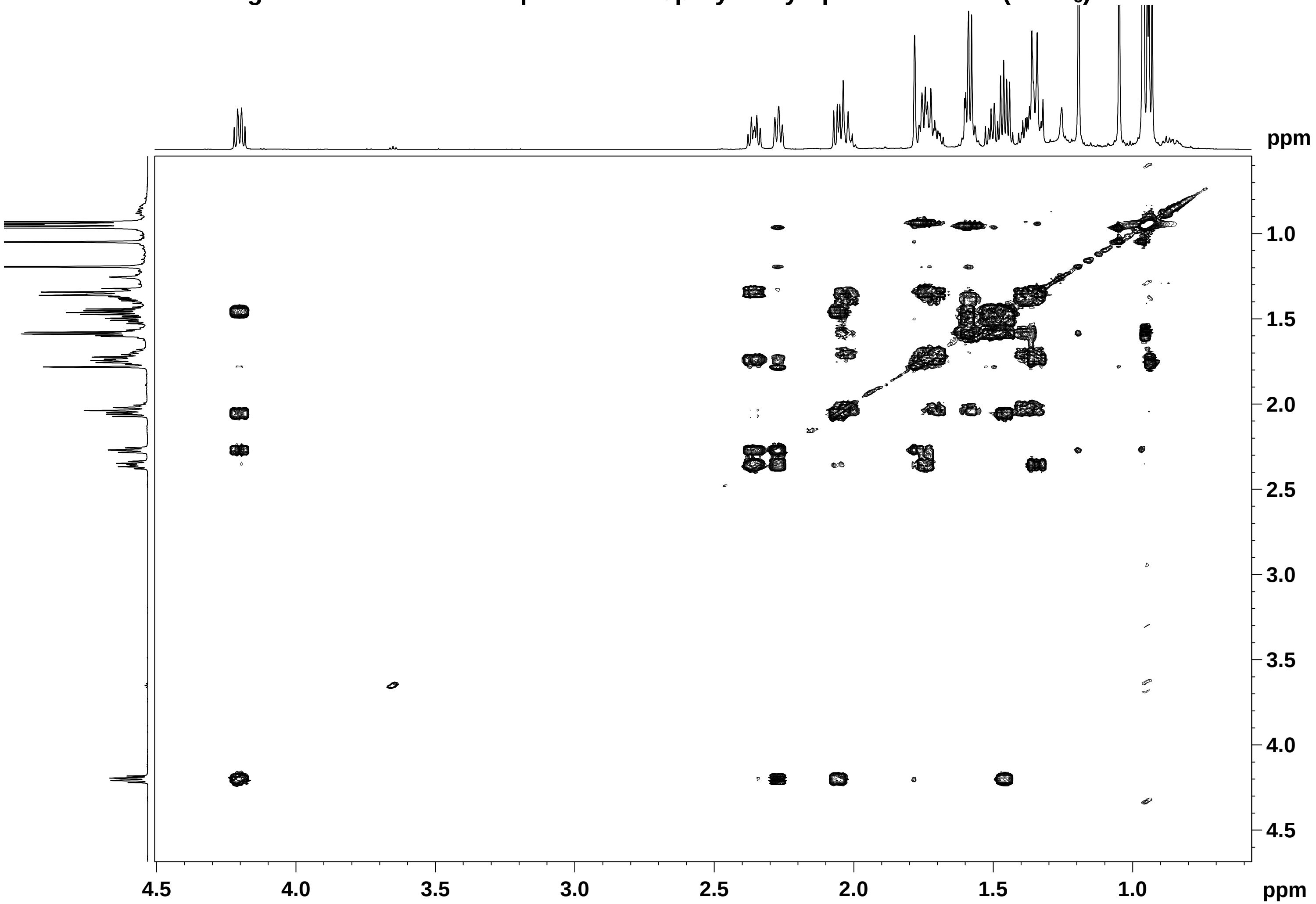

Figure 5e. HSQC spectrum of 9β-hydroxy-spiroviolane 11 ($CDCl_3$)

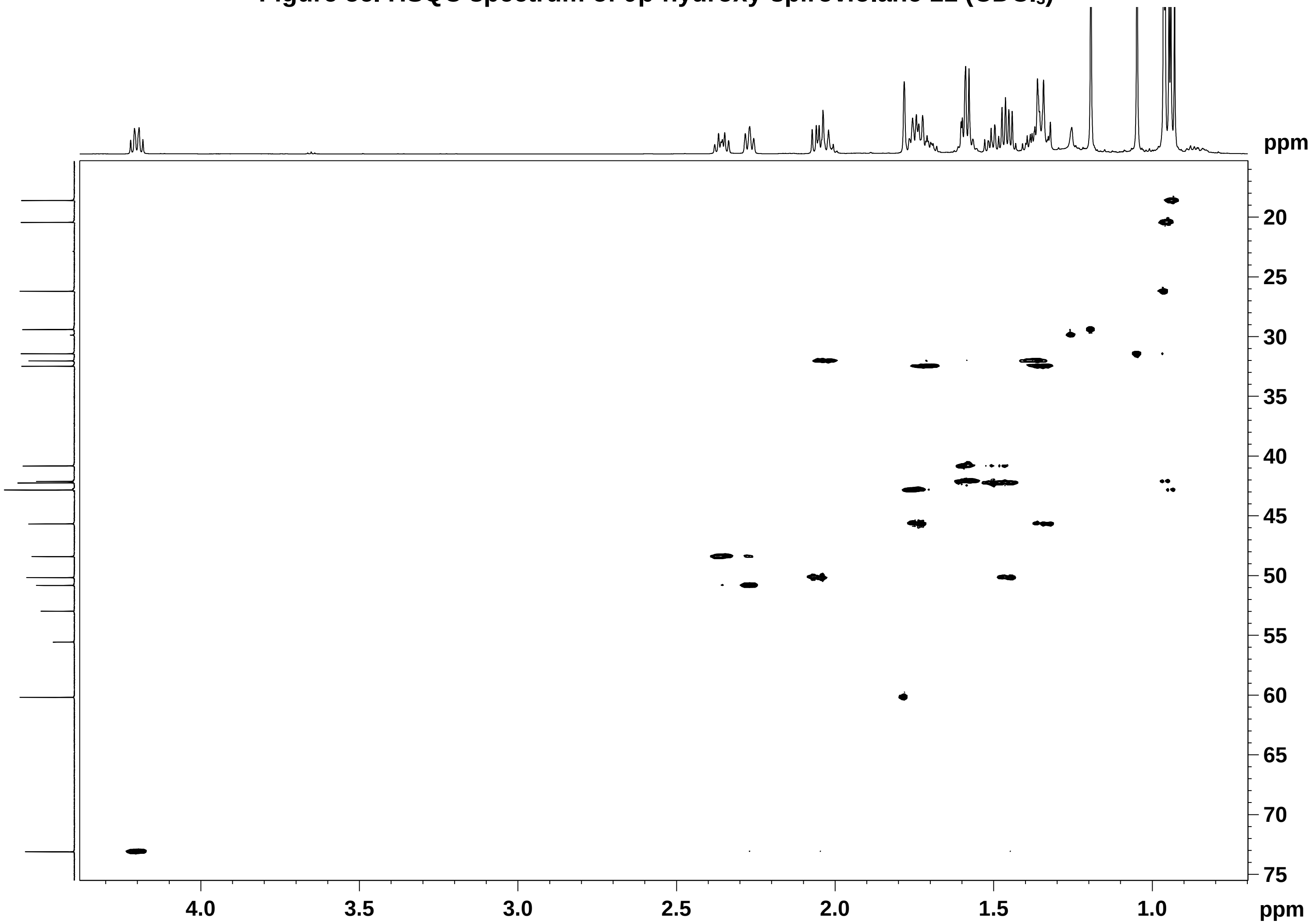

Figure 5f. HMBC spectrum of 9β-hydroxy-spiroviolane 11 ($CDCl_3$)

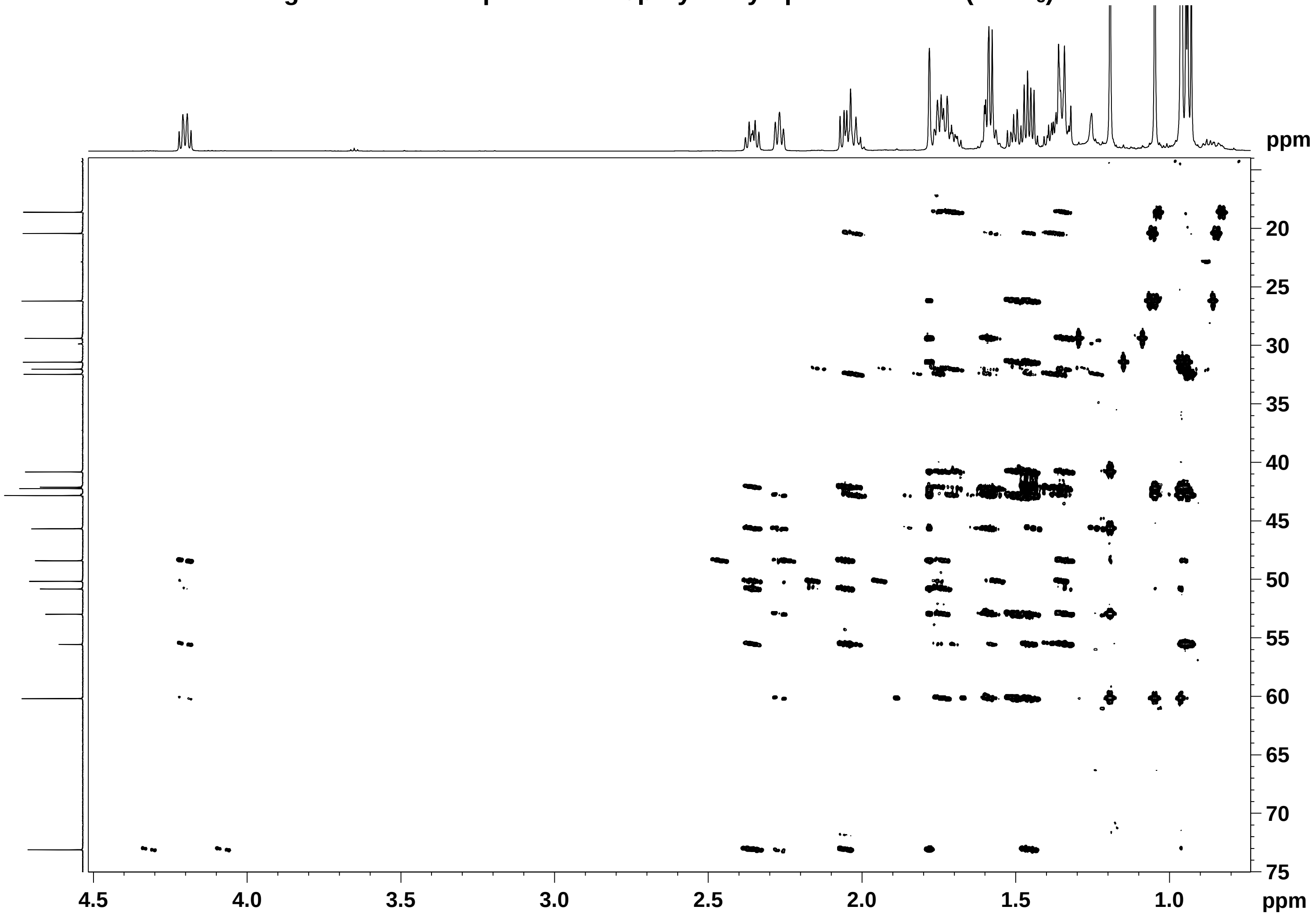

Figure 5g. NOESY spectrum of 9β-hydroxy-spiroviolane 11 ($CDCl_3$)

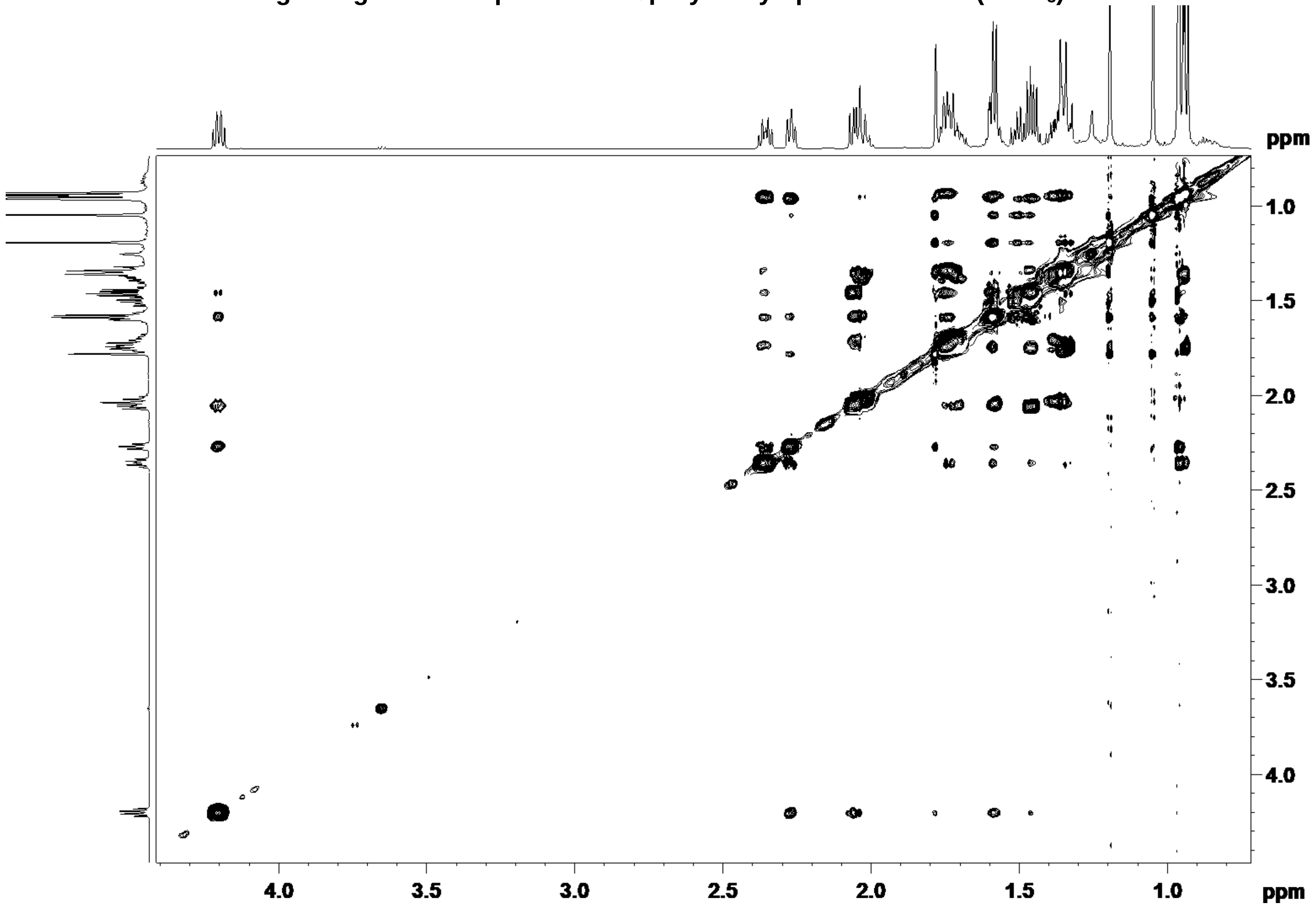

Figure 6a. ^{1}H NMR spectrum of 9α-hydroxy-spiroviolane 10 (600 MHz, $CDCl_3$)

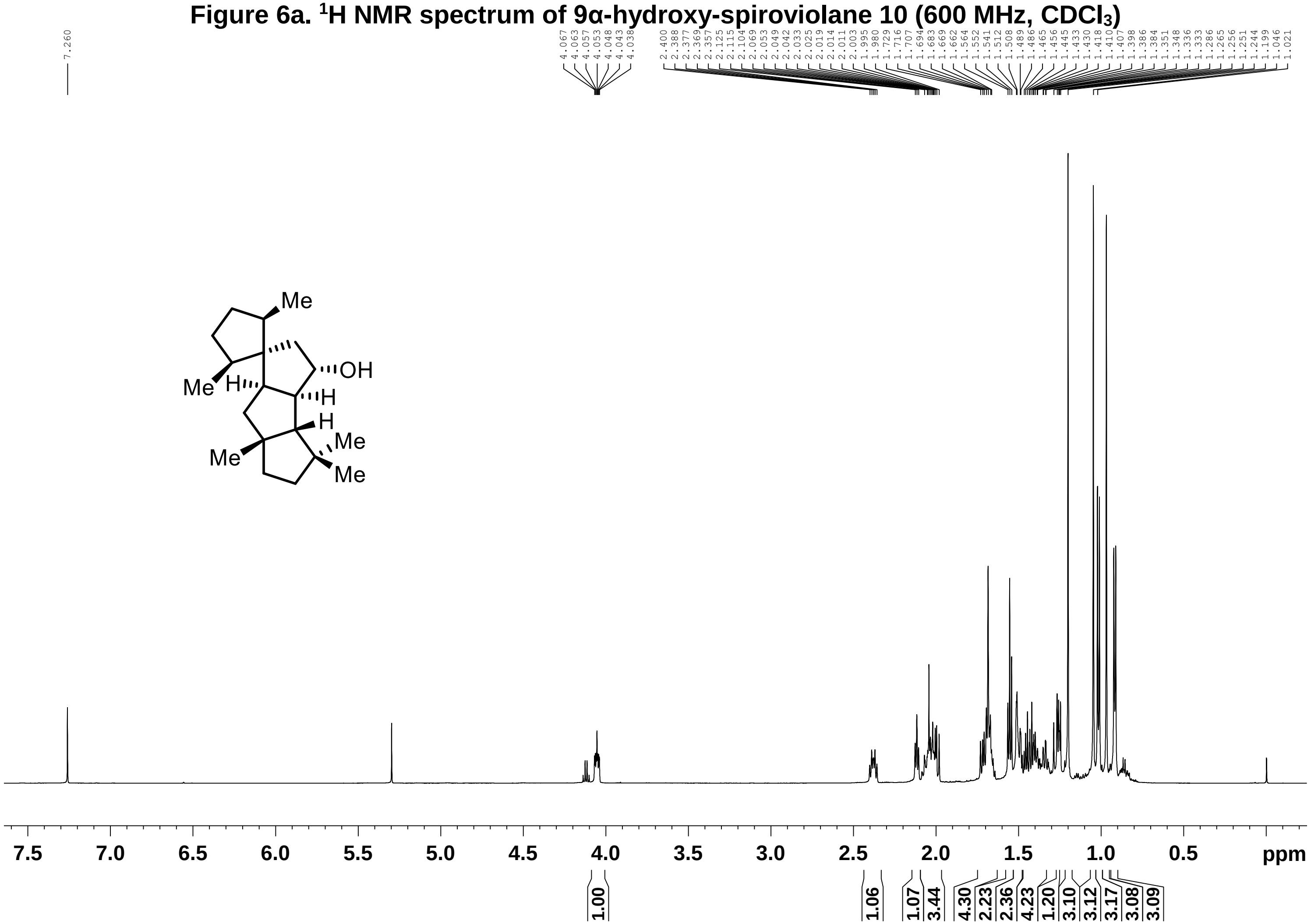

Figure 6b. ^{1}H NMR spectrum of 9α-hydroxy-spiroviolane 10 (600 MHz, CDCl$_3$)

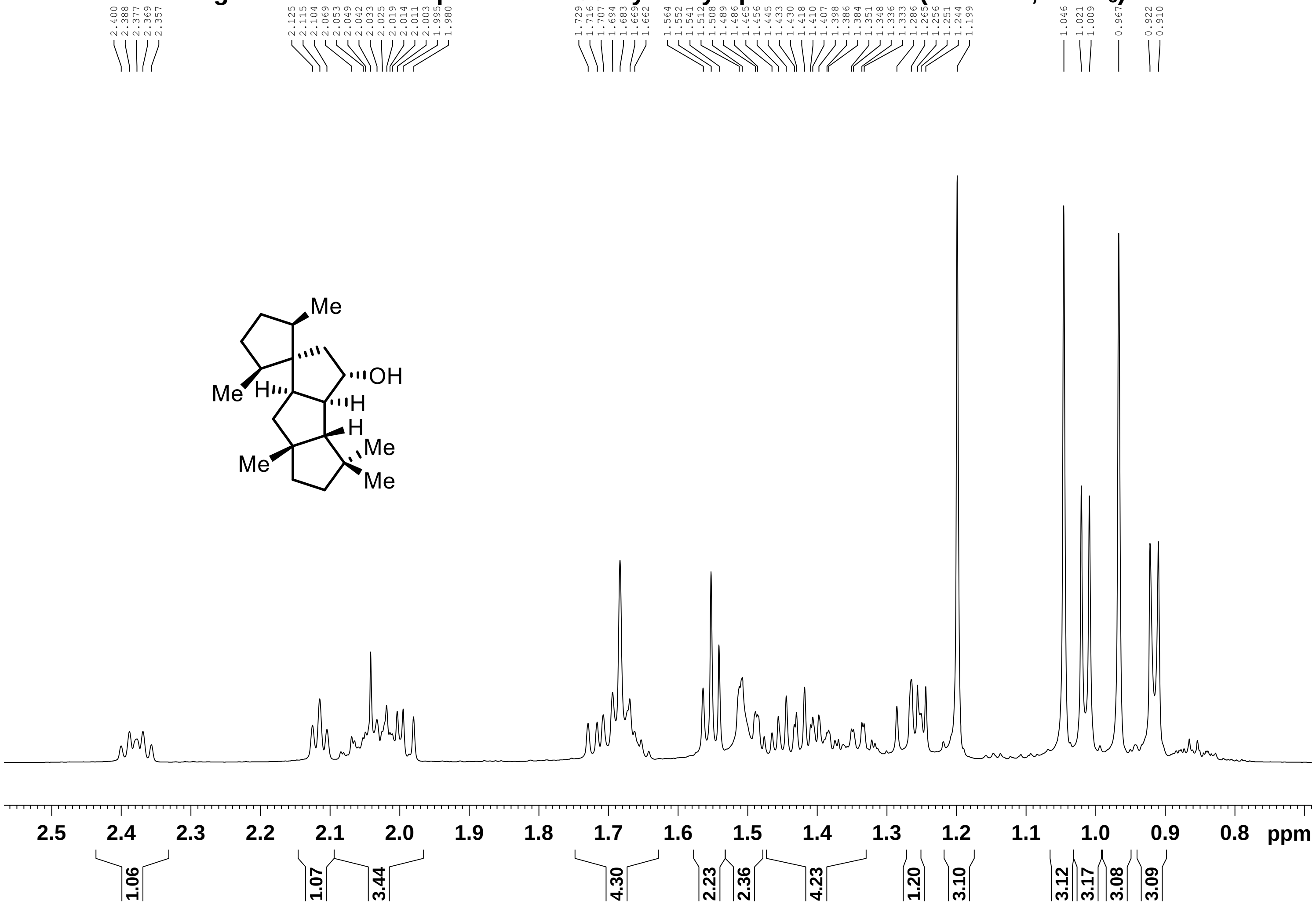

Figure 6c. ^{13}C NMR spectrum of 9α-hydroxy-spiroviolane 10 (150 MHz, $CDCl_3$)

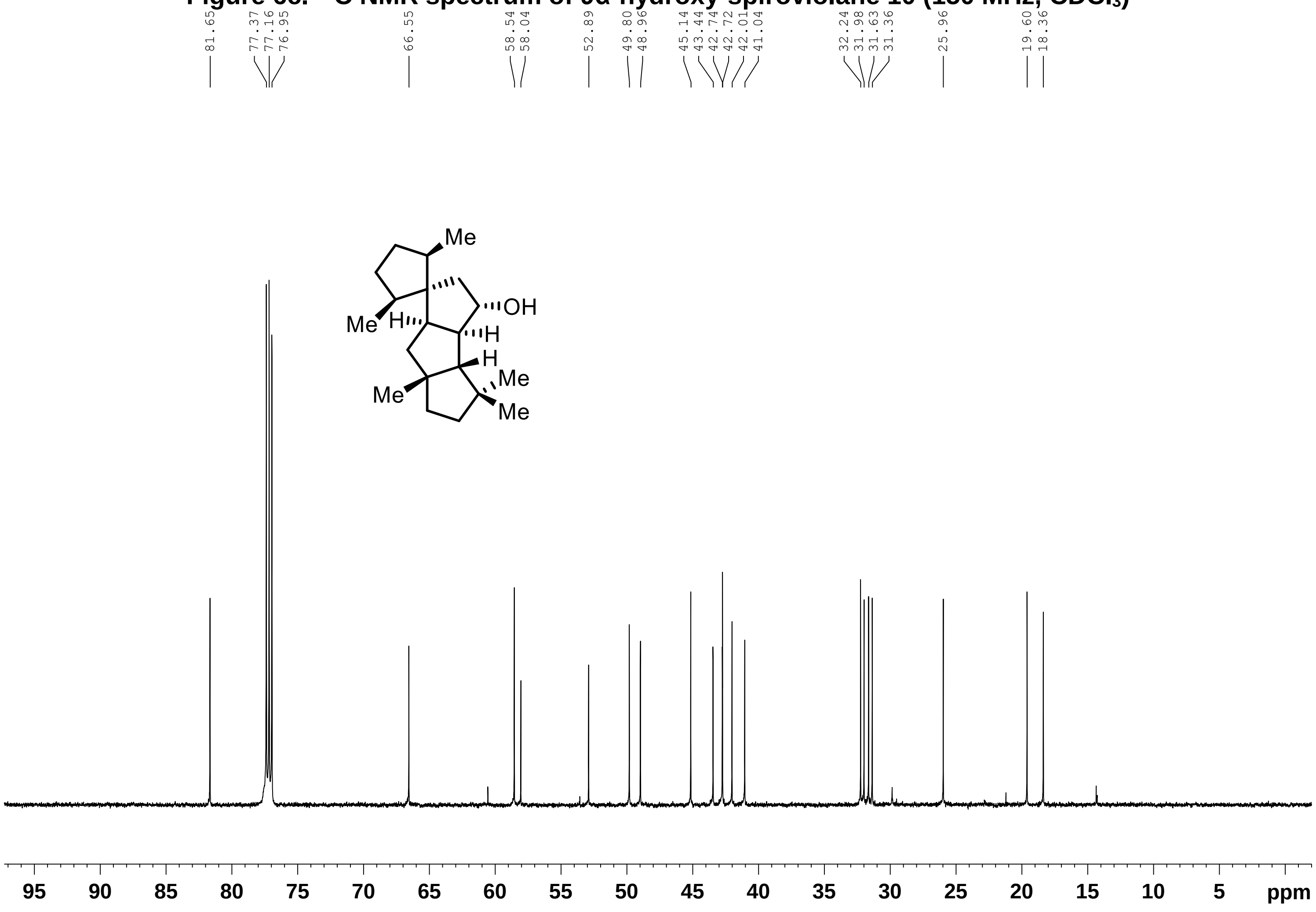

Figure 6d. ^1H-^1H COSY spectrum of 9α-hydroxy-spiroviolane 10 ($CDCl_3$)

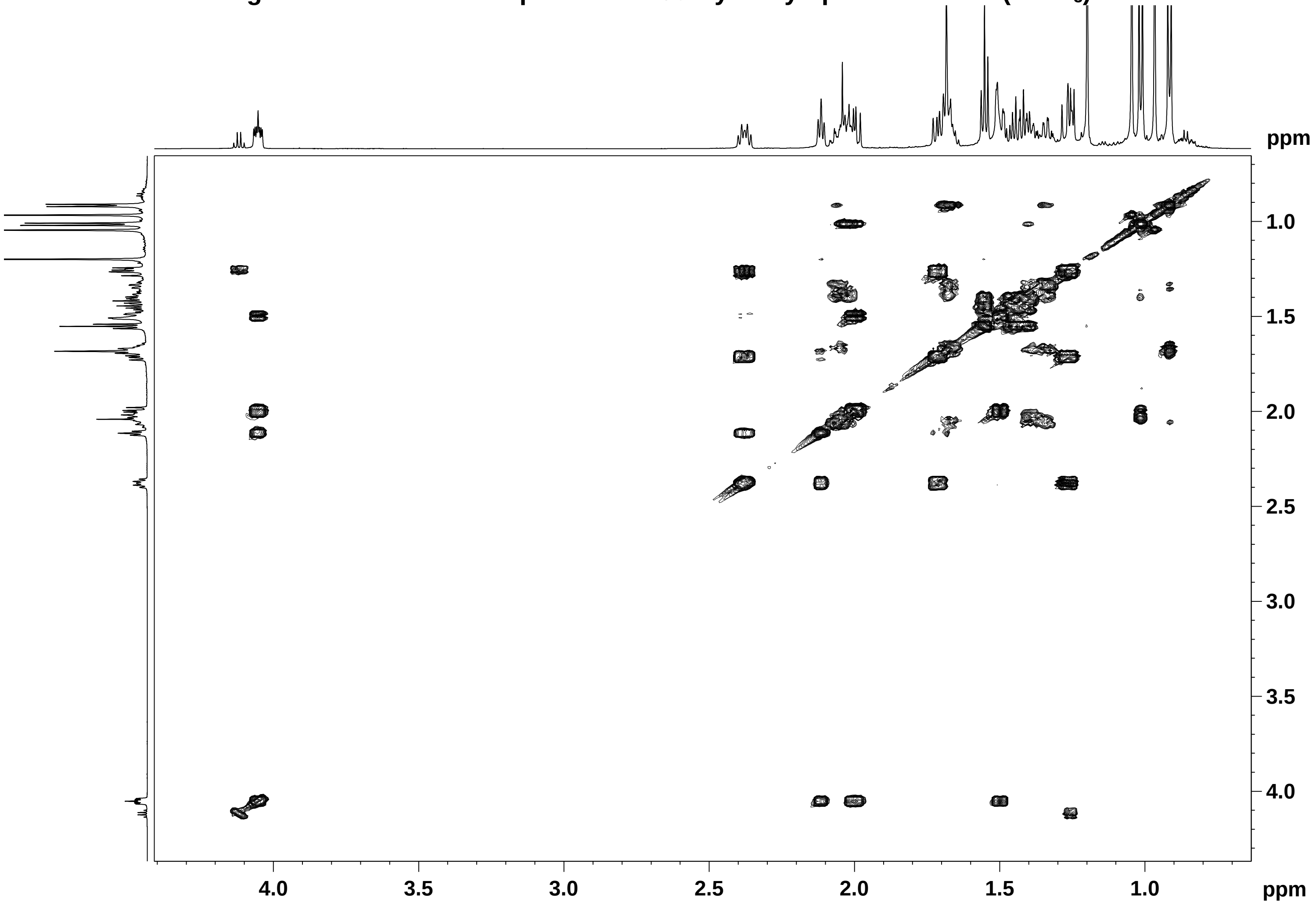

Figure 6e. HSQC spectrum of 9α-hydroxy-spiroviolane 10 ($CDCl_3$)

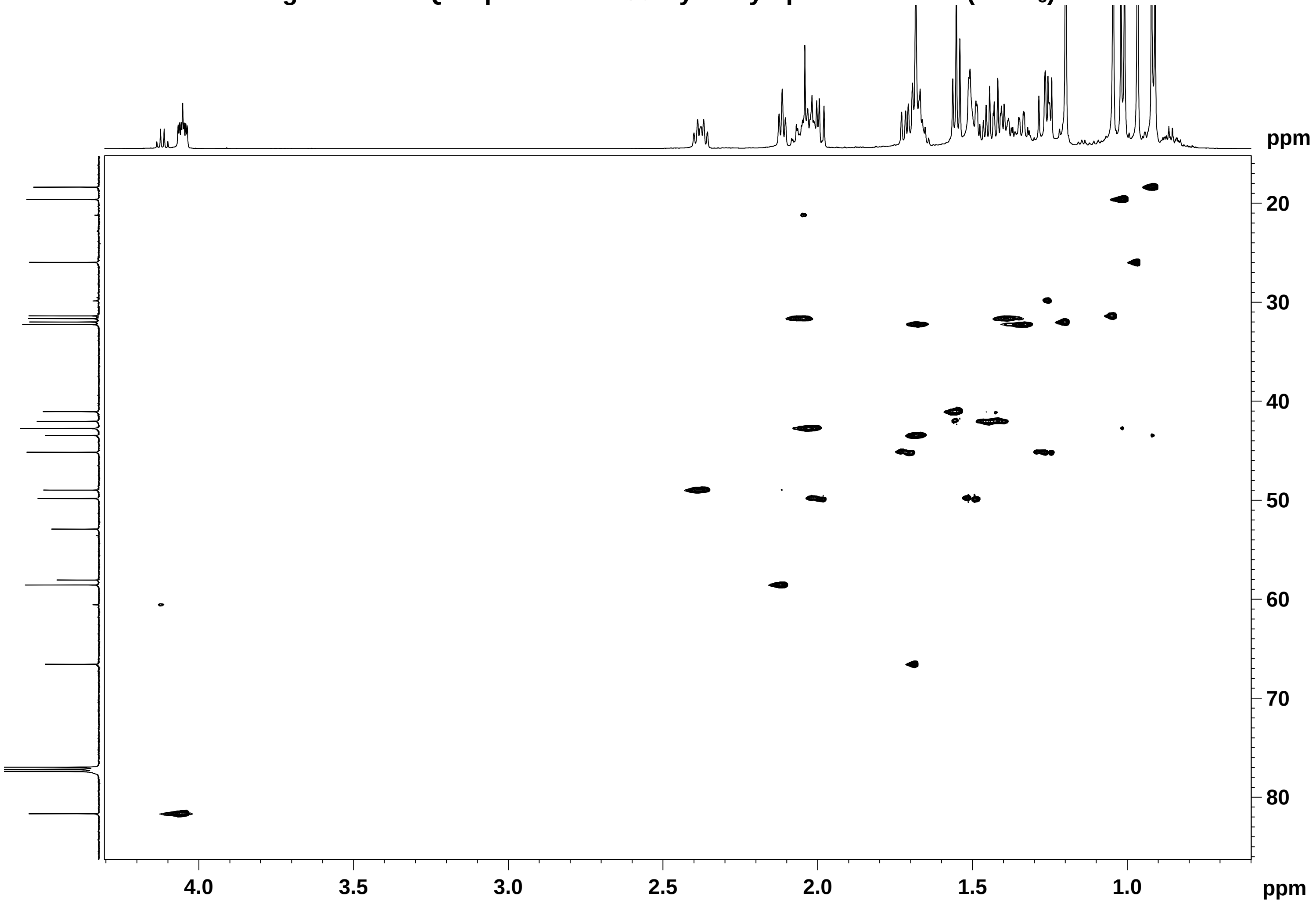

Figure 6f. HMBC spectrum of 9α-hydroxy-spiroviolane 10 ($CDCl_3$)

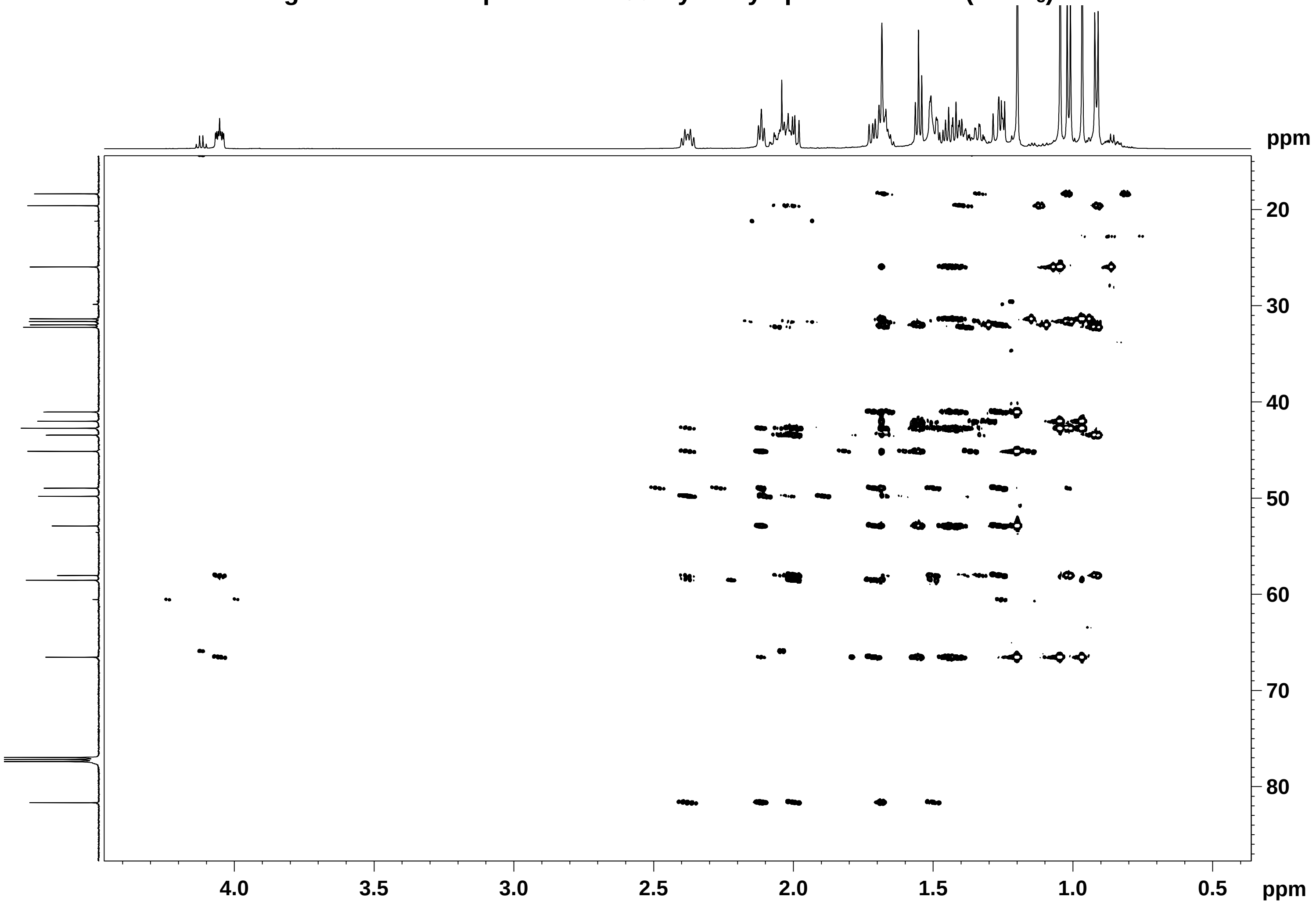

Figure 6g. NOESY spectrum of 9α-hydroxy-spiroviolane 10 ($CDCl_3$)

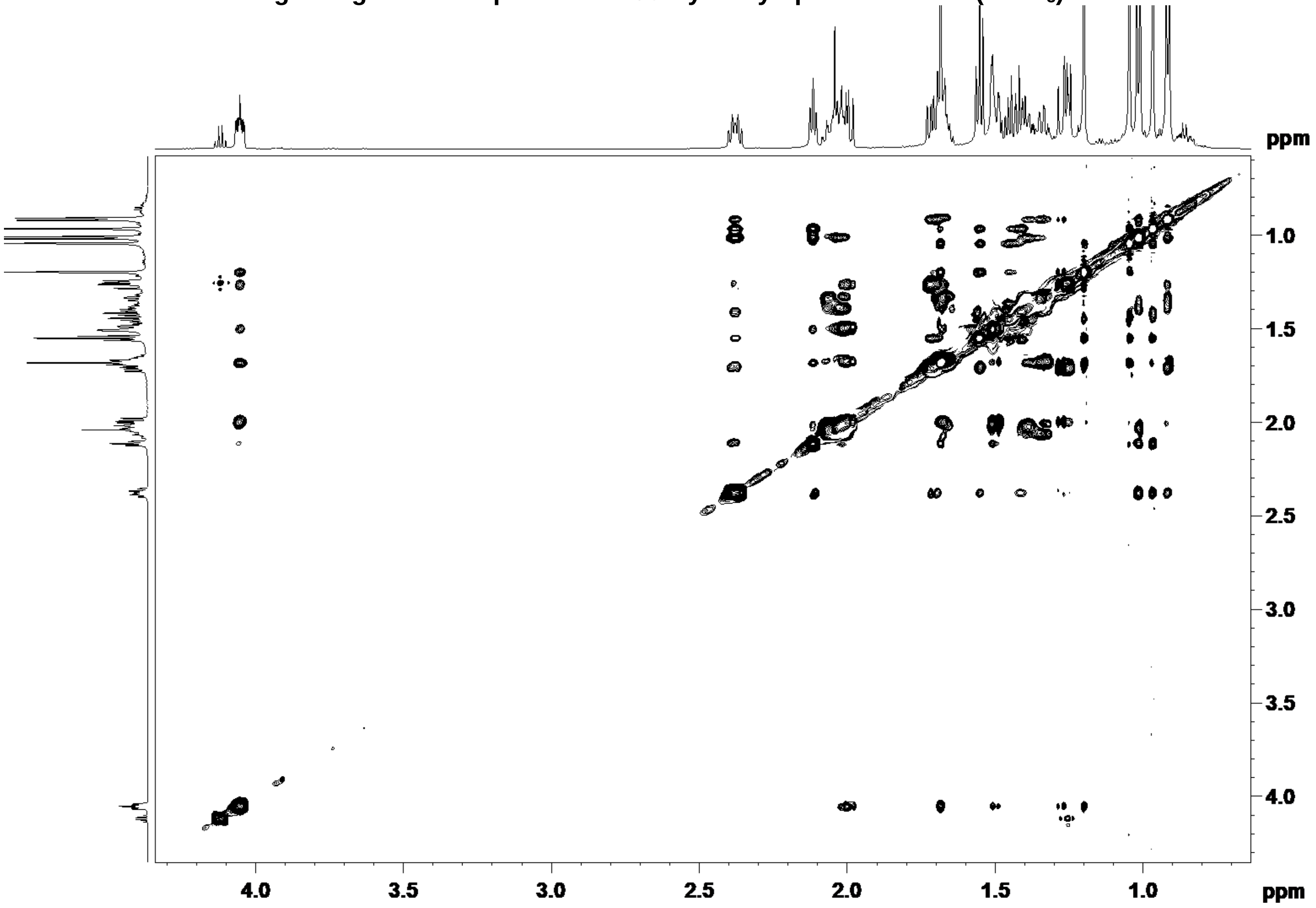

Figure 7a. ¹H NMR spectrum of compound 9-oxo-spiroviolane 12 (400 MHz, $CDCl_3$)

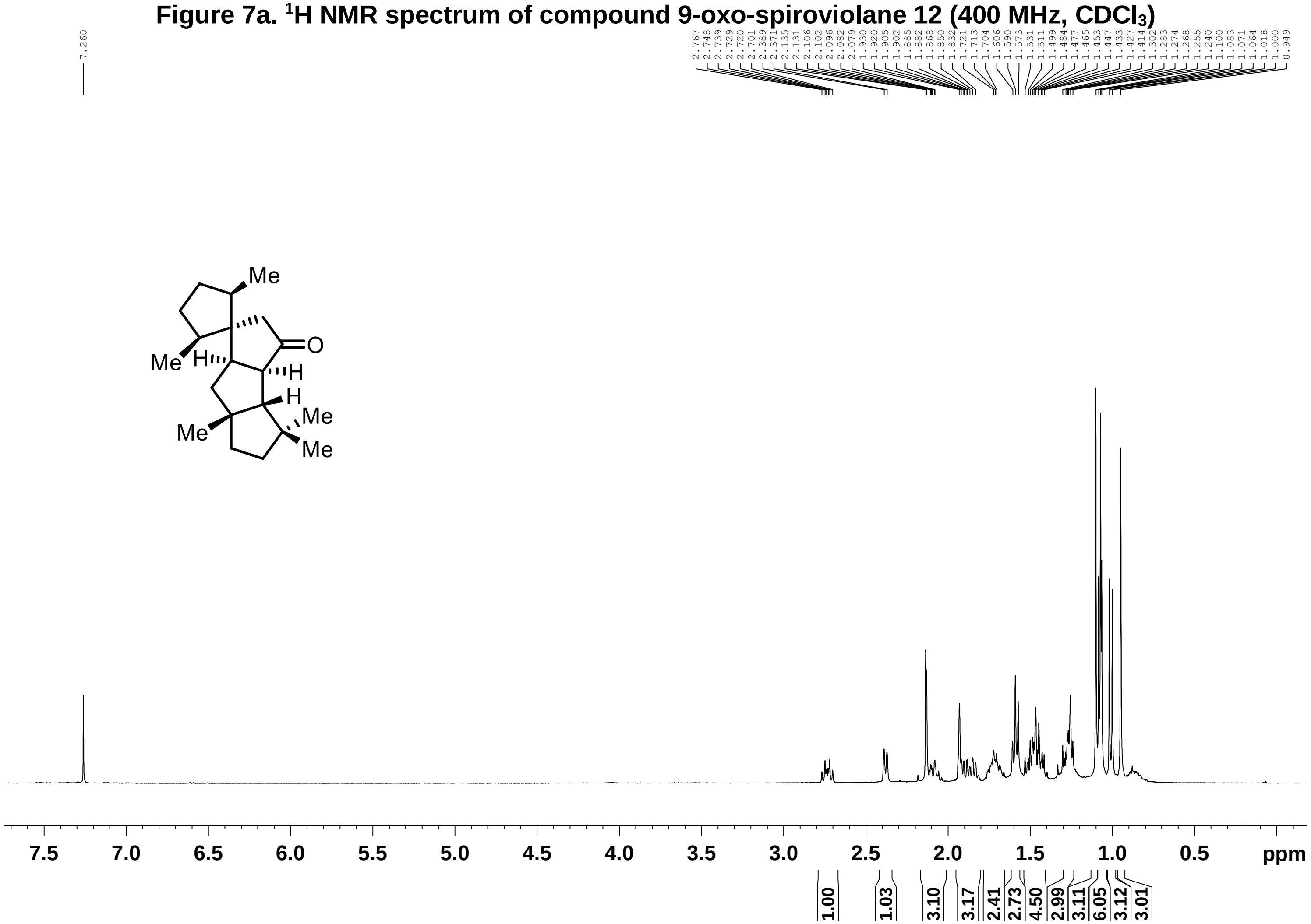

Figure 7b. ^1H NMR spectrum of compound 9-oxo-spiroviolane 12 (400 MHz, $CDCl_3$)

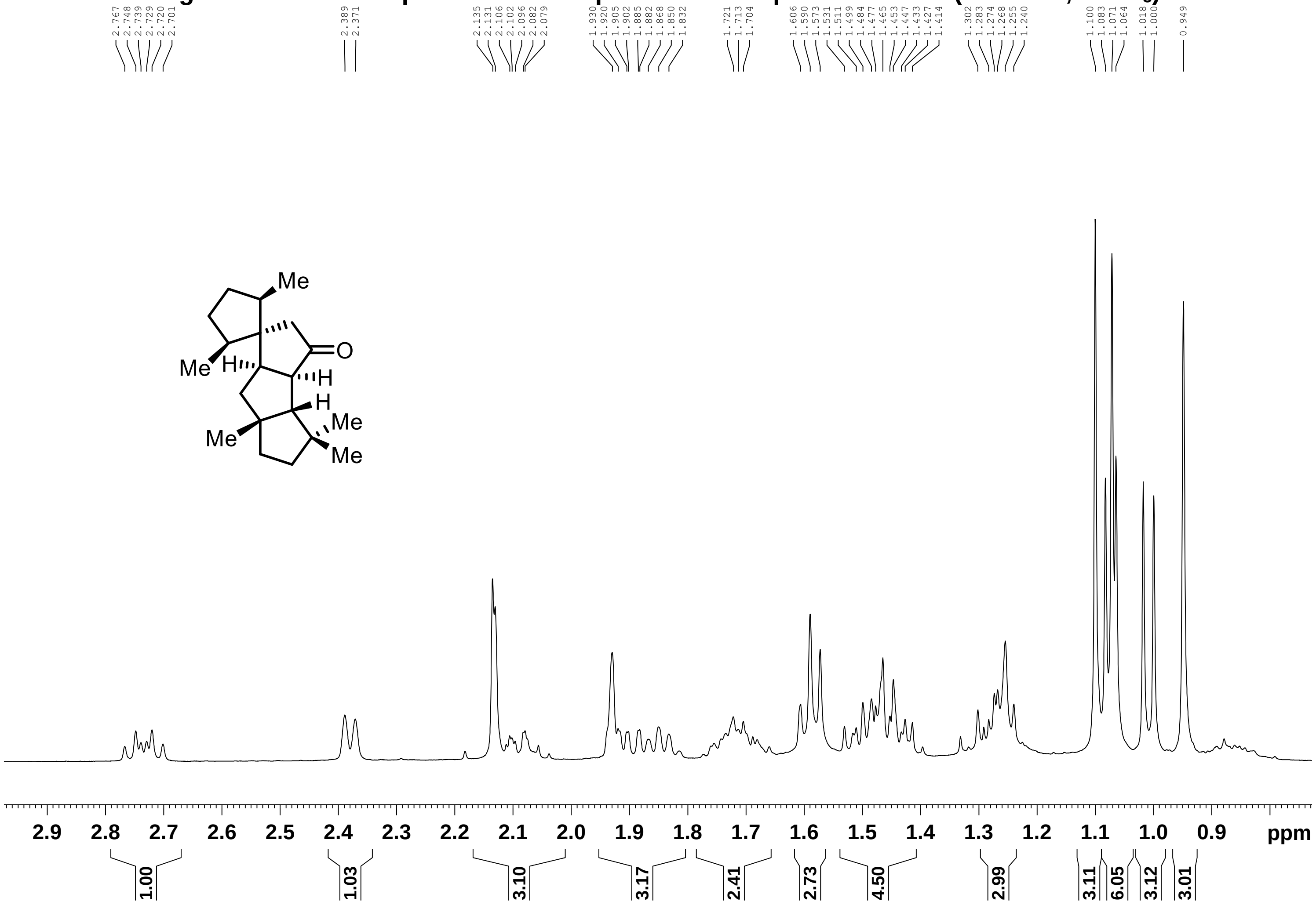

Figure 7c. ^{13}C NMR spectrum of 9-oxo-Spiroviolane 12 (100 MHz, $CDCl_3$)

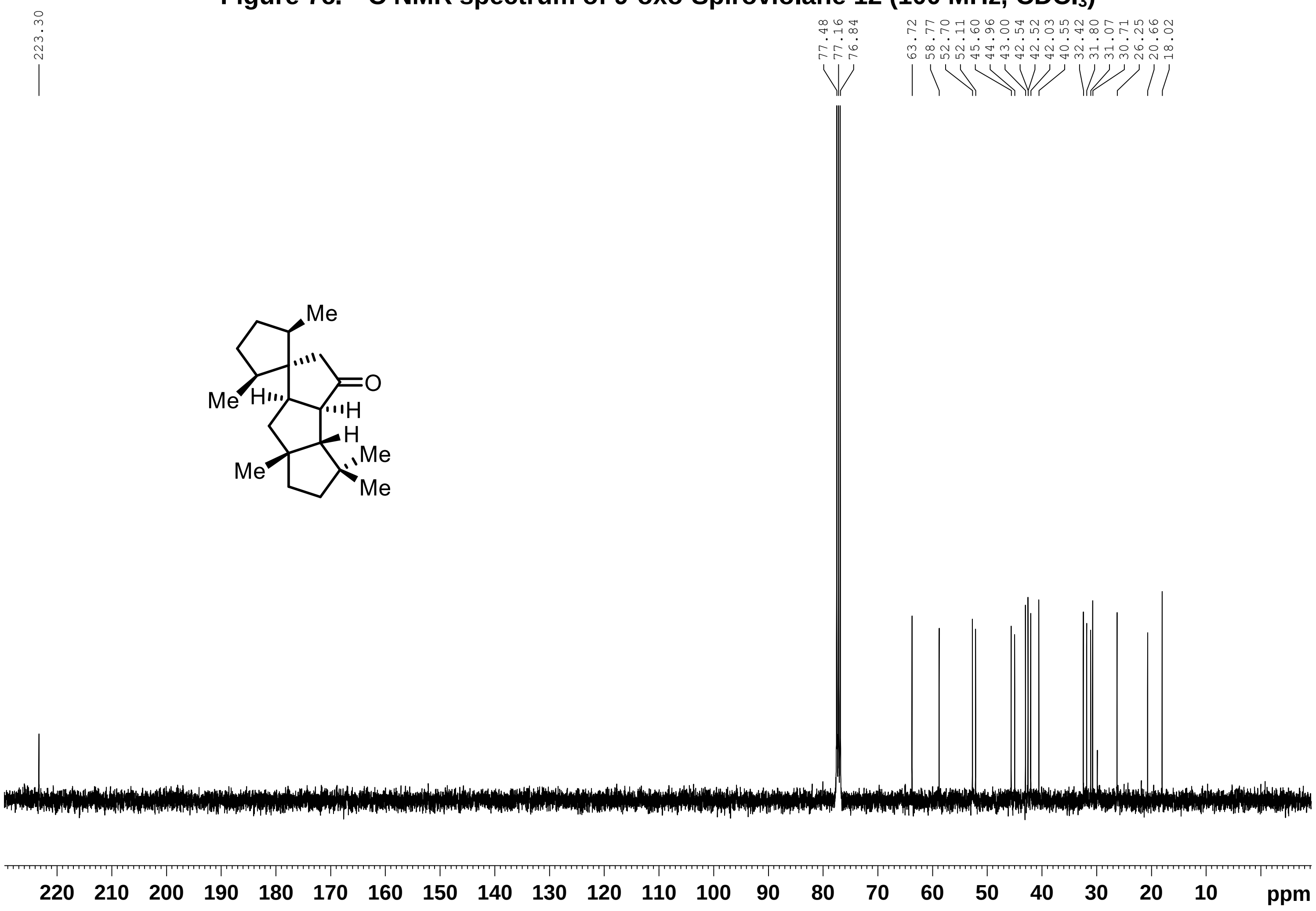

Figure 8a. ^{1}H NMR spectrum of compound 13 (400 MHz, $CDCl_3$)

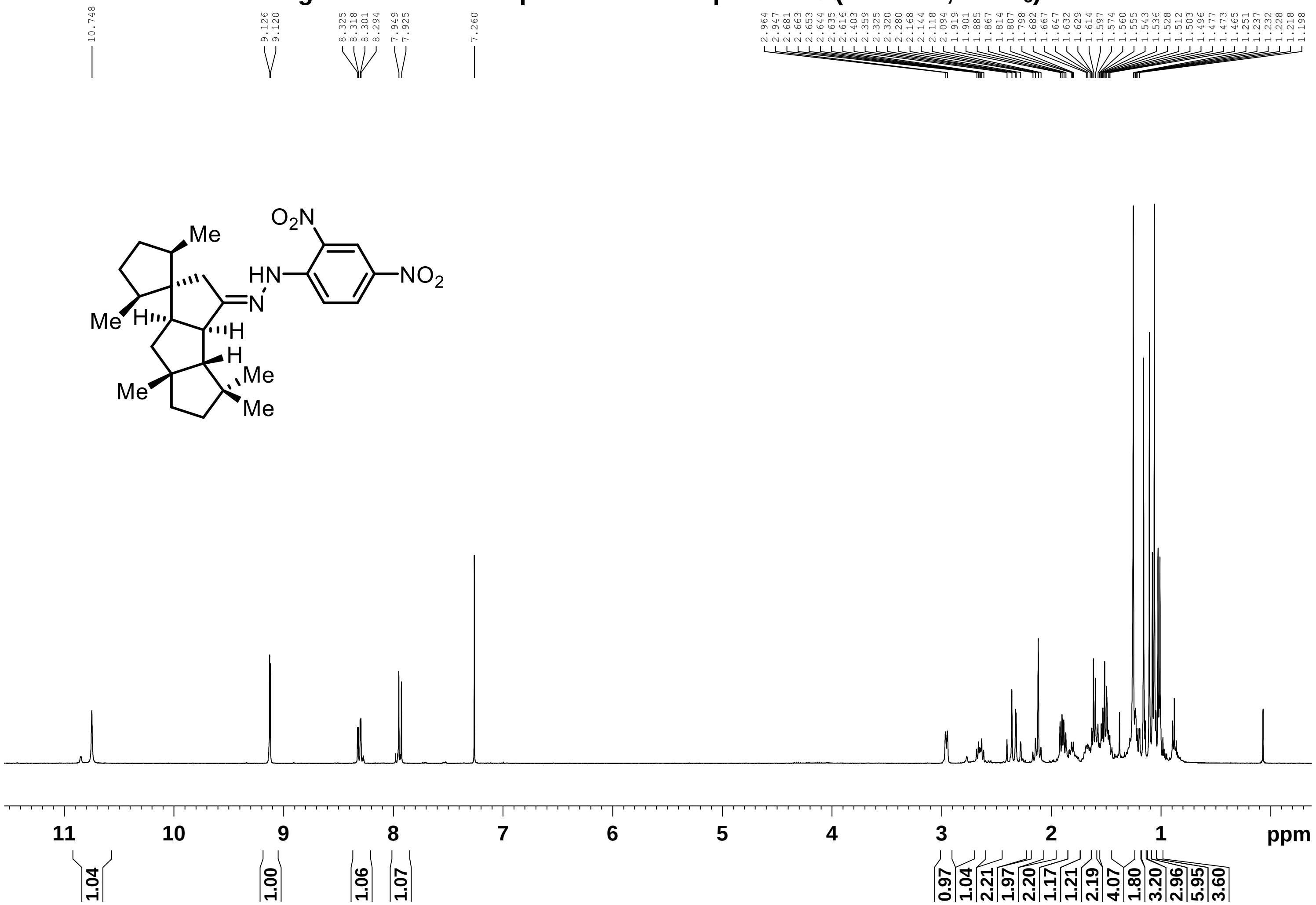

Figure 8b. ¹H NMR spectrum of compound 13 (400 MHz, $CDCl_3$)

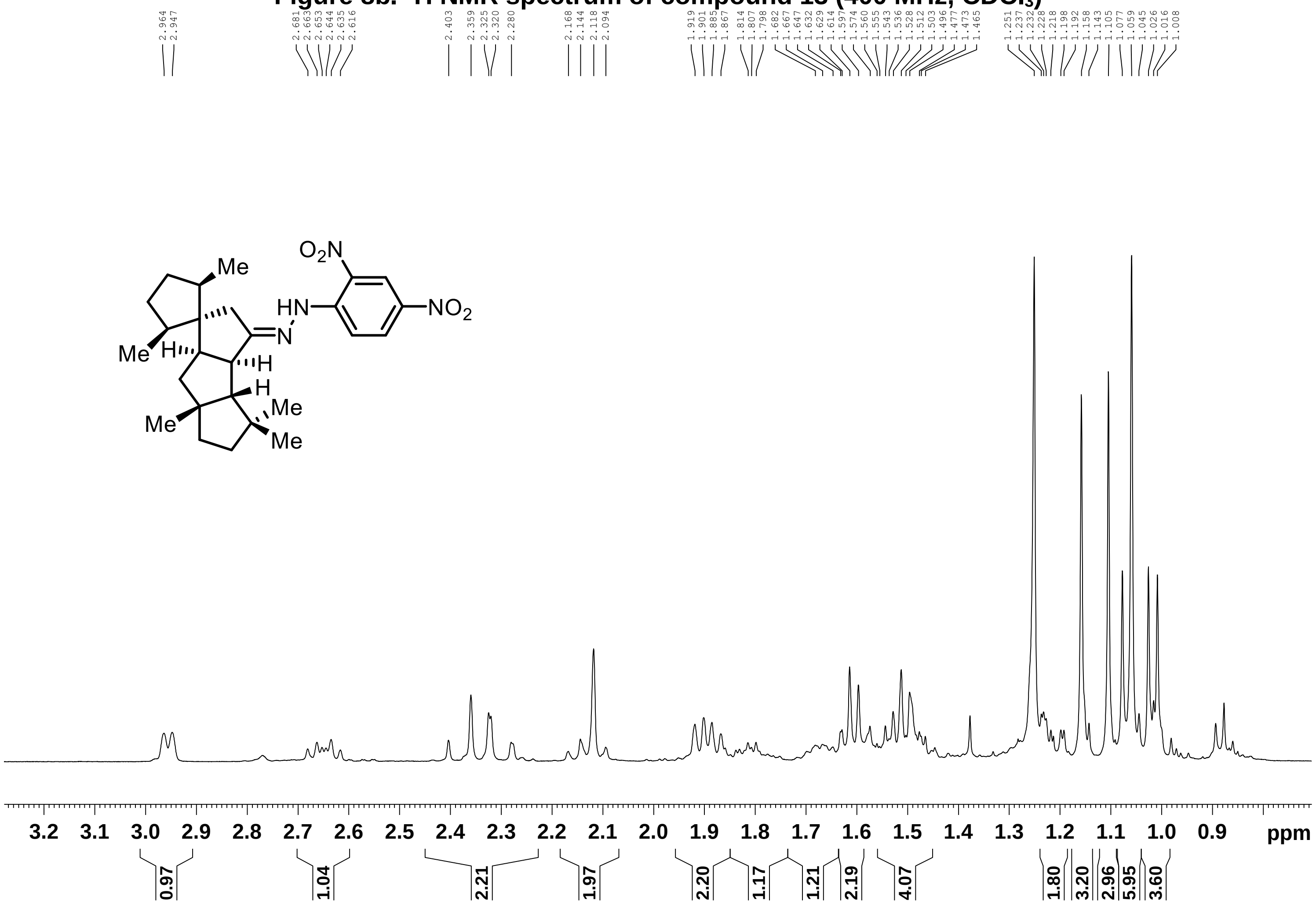

Figure 8c. ^{13}C NMR spectrum of compound 13 (100 MHz, $CDCl_3$)

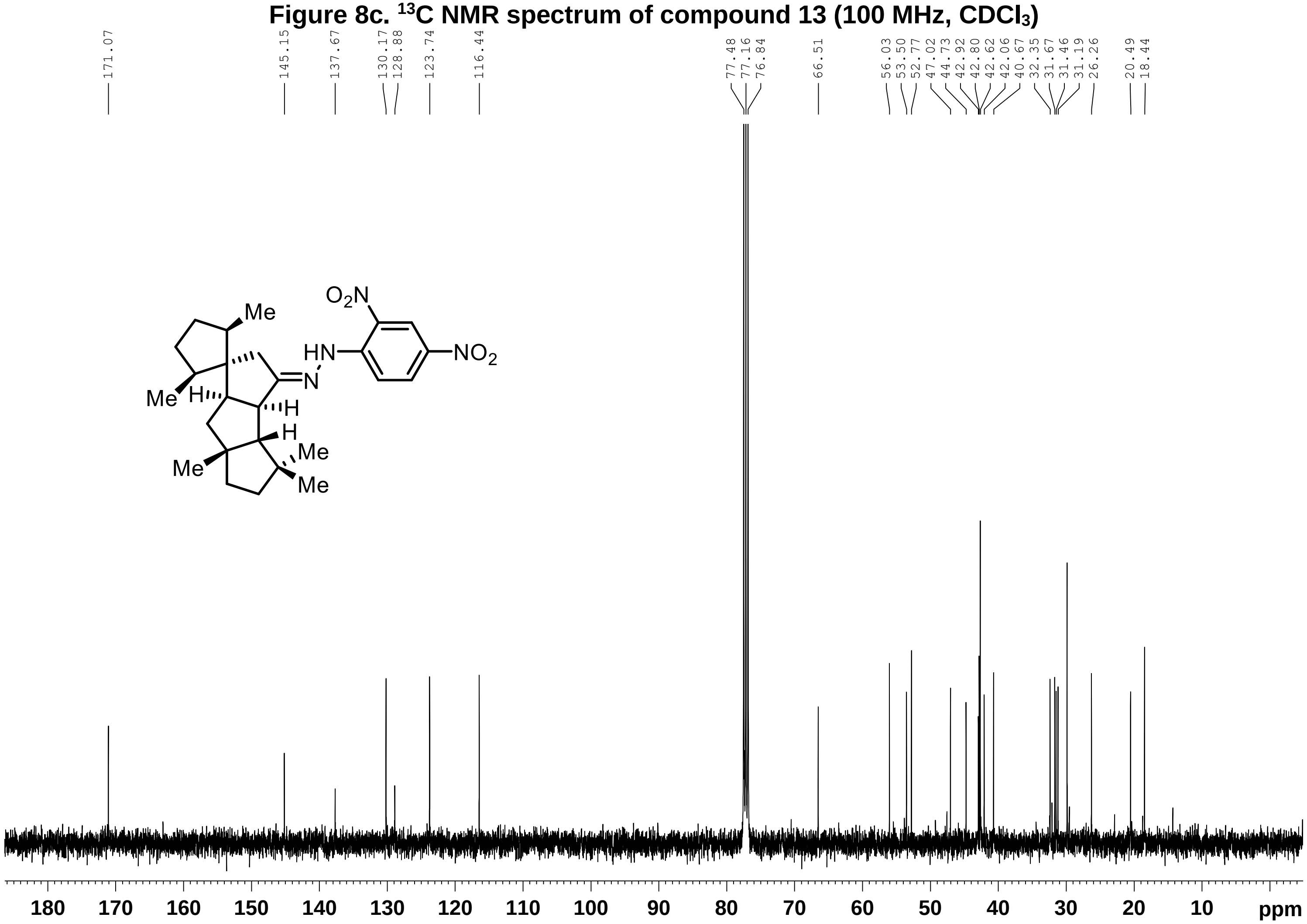

References

1. Kieser, T.; Bibb, M. J.; Buttner, M. J.; Chater, K. F.; Hopwood, D. A. Practical Streptomyces genetics. The John Innes Foundation, Norwich. **2000**.
2. Agilent Technologies, CrysAlisPro, Version 1.171.37.35. Yarnton, Oxfordshire, United Kindom, **2014**.
3. Dolomanov, O. V.; Bourhis, L. J.; Gildea, R. J.; Howard, J. A. K.; Puschmann, H. OLEX2: a complete structure solution, refinement and analysis program. *J. Appl. Cryst*. **2009**, *42*, 339-341.
4. *Sheldrick, G. M.* Crystal structure refinement with SHELXL. *Acta Crystallogr. C,* **2015**, 71, 3-8.
5. Rabe, P.; Rinkel, J.; Dolja, E.; Schmitz, T.; Nubbemeyer, B.; Luu, T. H.; Dickschat, J. S. Mechanistic investigations of two bacterial diterpene cyclases: spiroviolene synthase and tsukubadiene synthase. *Angew. Chem. Int. Ed.* **2017**, *56*, 2776-2779.
6. Chi, H. M.; Cole, C. J. F.; Hu, P.; Taylor, C. A.; Snyder, S. A. Total syntheses of spiroviolene and spirograterpene A: a structural reassignment with biosynthetic implications. *Chem. Sci.* **2020**, *11*, 10939-10944.
7. Hearn, M. J.; Lebold, S. A.; Sinha, A.; Sy, K. Preparation and absorption spectra of arylhydrazones from α,β-unsaturated carbonyl compounds. *J. Org. Chem.* **1989**, *54*, 4188-4193.
8. Xu, H.; Dickschat, J. S. Revision of the cyclisation mechanism for the diterpene spiroviolene and investigations of its mass spectrometric fragmentation. *ChemBioChem* **2021**, *22*, 850-854.
